# Omics analysis coupled with gene editing revealed potential transporters and regulators related to levoglucosan metabolism efficiency of the engineered *Escherichia coli*

**DOI:** 10.1186/s13068-022-02102-4

**Published:** 2022-01-11

**Authors:** Dongdong Chang, Cong Wang, Zia Ul Islam, Zhisheng Yu

**Affiliations:** 1 College of Resources and Environment, University of Chinese Academy of Sciences, Beijing, 100049 People’s Republic of China; 2 Department of Chemistry, Mississippi State University, Starkville, MS 39762 USA; 3 RCEES-IMCAS-UCAS Joint-Lab of Microbial Technology for Environmental Science, Beijing, 100085 People’s Republic of China

**Keywords:** Levoglucosan, Proteomics, Transporter, Regulator, Bioconversion, CRISPR/Cas9

## Abstract

**Background:**

Bioconversion of levoglucosan, a promising sugar derived from the pyrolysis of lignocellulose, into biofuels and chemicals can reduce our dependence on fossil-based raw materials. However, this bioconversion process in microbial strains is challenging due to the lack of catalytic enzyme relevant to levoglucosan metabolism, narrow production ranges of the native strains, poor cellular transport rate of levoglucosan, and inhibition of levoglucosan metabolism by other sugars co-existing in the lignocellulose pyrolysate. The heterologous expression of eukaryotic levoglucosan kinase gene in suitable microbial hosts like *Escherichia coli* could overcome the first two challenges to some extent; however, no research has been dedicated to resolving the last two issues till now.

**Results:**

Aiming to resolve the two unsolved problems, we revealed that seven ABC transporters (XylF, MalE, UgpB, UgpC, YtfQ, YphF, and MglA), three MFS transporters (KgtP, GntT, and ActP), and seven regulatory proteins (GalS, MhpR, YkgD, Rsd, Ybl162, MalM, and IraP) in the previously engineered levoglucosan-utilizing and ethanol-producing *E. coli* LGE2 were induced upon exposure to levoglucosan using comparative proteomics technique, indicating these transporters and regulators were involved in the transport and metabolic regulation of levoglucosan. The proteomics results were further verified by transcriptional analysis of 16 randomly selected genes. Subsequent gene knockout and complementation tests revealed that ABC transporter XylF was likely to be a levoglucosan transporter. Molecular docking showed that levoglucosan can bind to the active pocket of XylF by seven H-bonds with relatively strong strength.

**Conclusion:**

This study focusing on the omics discrepancies between the utilization of levoglucosan and non-levoglucosan sugar, could provide better understanding of levoglucosan transport and metabolism mechanisms by identifying the transporters and regulators related to the uptake and regulation of levoglucosan metabolism. The protein database generated from this study could be used for further screening and characterization of the transporter(s) and regulator(s) for downstream enzymatic/genetic engineering work, thereby facilitating more efficient microbial utilization of levoglucosan for biofuels and chemicals production in future.

**Supplementary Information:**

The online version contains supplementary material available at 10.1186/s13068-022-02102-4.

## Background

The increasing concerns on global energy crisis and climate change have prompted the development of renewable and sustainable resources for biofuels and chemicals production as an alternative to traditional fossil-based fuels and chemicals. Lignocellulosic biomass, as the most abundant and non-food-oriented resource generated from solar energy and carbon dioxide fixation on our planet, is environment-friendly and renewable, and has been researched extensively in past decades [1]. However, it is still somewhat problematic for the efficient utilization of lignocellulosic biomass, which requires two main steps: (1) depolymerization of the lignocellulose into fermentable sugars by pretreatment procedures and (2) bioconversion of sugars by microbial fermentation. One of the major challenges involved in this conversion process is the lack of fermentative microorganisms that could effectively utilize the non-glucose lignocellulose-derived substrates, such as levoglucosan [2].

Levoglucosan is an abundant sugar present in the lignocellulosic pyrolysate produced by pyrolysis technique [3], which takes the lowest capital cost among all the biomass pretreatment processes [4]. Therefore, levoglucosan is considered a promising renewable resource for producing biofuels and chemicals. In nature, a few native microorganisms could metabolize levoglucosan [3, 5–10]; however, their productions with narrow range and low value greatly limit their application as the fermenting strains to produce valuable products. Eukaryotic levoglucosan kinase (LGK) from fungi and yeast [6, 8, 9] and prokaryotic levoglucosan dehydrogenase (LGDH) from bacteria [10, 11] were found responsible for levoglucosan assimilation, thereby laying the foundation for the downstream engineering work on targeted bioconversion of levoglucosan. Recently, LGK catalyzing the phosphorylation of levoglucosan found in *Lipomyces starkeyi* YZ215 was cloned [12] and heterologously expressed in some platform bacteria to produce various biofuels and chemicals [13–17]. Nevertheless, the poorly known transmembrane transport of levoglucosan, which is the first key limiting step for microbial utilization of levoglucosan, could limit the downstream pathway flux to a great extent [15, 18], resulting in a longer lag phase and lower product productivity during levoglucosan fermentation than glucose [16] and fructose [17] fermentations. In addition, during biomass pyrolysis, a maximum of 2.9 wt% fructose can be coproduced with levoglucosan [3]. Levoglucosan metabolism is severely repressed by other carbon sources like glucose and fructose [17, 19] through the carbon catabolite repression (CCR) effect, which allows cells utilize the most energy-efficient carbon source in a sugar mixture and thus leads to a diauxic growth that limits the conversion efficiency of levoglucosan during the co-fermentation process [15, 19, 20]. Therefore, understanding and revealing the proteins related to the transport and CCR of levoglucosan are crucial for enhancing the levoglucosan conversion efficiency and cell growth rate.

Global proteomics has shown promise for the discovery of proteins with currently unrecognized functions [21]. With regard to the cells exposed to different physiological cues, comparative proteomics can serve as a unique and informative “readout” of two different physiological states, enabling the unraveling of the molecular mechanisms involved in a certain biological process [22]. Moreover, by providing an overview of the entire biochemical pathways, proteomics profiling can complement and extend our knowledge regarding the biological roles of proteins, especially, the newly identified differentially expressed proteins (DEPs). Hence, proteomics could help us discover the potentially crucial proteins involved in levoglucosan transport and catabolite repression, thereby aiding enzyme and metabolic engineering to facilitate enhanced levoglucosan uptake and metabolism efficiency, ultimately yielding improved production of biofuels and chemicals from levoglucosan.

In this study, a previously engineered levoglucosan-utilizing ethanologenic *Escherichia coli* strain [17] was grown in the M9 minimal media containing either levoglucosan or fructose and harvested at both early- and mid-log phases. Comparison of the proteomics of levoglucosan-feeding cells with that of fructose-feeding cells revealed remarkable differences in the protein content of these cells. The changes in protein content of these cells might reflect a variety of proteins involved in levoglucosan transport, metabolism, and metabolic regulation. To the best of our knowledge, this is the first study focusing on the biomolecular discrepancies between the cellular metabolism of levoglucosan and another sugar and identifying proteins related to the transport and CCR of levoglucosan. The understanding of the levoglucosan transport and metabolism mechanism and the proteins involved in it could produce considerable datasets and resources to facilitate future research on efficient microbial utilization of levoglucosan for biofuels and chemicals with better yields at pilot and industrial scales.

## Results and discussion

*Escherichia coli* is a commonly used platform microorganism that possesses native transporters and metabolism pathways for many sugar substrates, while it innately cannot utilize levoglucosan [3]. Levoglucosan can be metabolized to glucose-6-phosphate (G6P) by genetically engineered *E. coli* in which LGK is heterologously expressed [17]; however, by what transporters levoglucosan is transported into cell cytoplasm and by what biomolecules this transport and metabolism process is regulated, are still not clear. Thus, a global insight into the cellular proteomics changes during the uptake and consumption of levoglucosan coupled with other validation work like transcriptional analysis and gene knockout could certainly provide clues about the transport and metabolic regulation of levoglucosan, providing a theoretical basis for engineering more robust levoglucosan-utilizing strains in pyrolysis-based biorefineries.

### Overview of the DEPs by COG, GO, and KEGG analysis revealed the DEPs were mainly involved in carbohydrate transport, localization, and metabolism

**Fig. 1:**
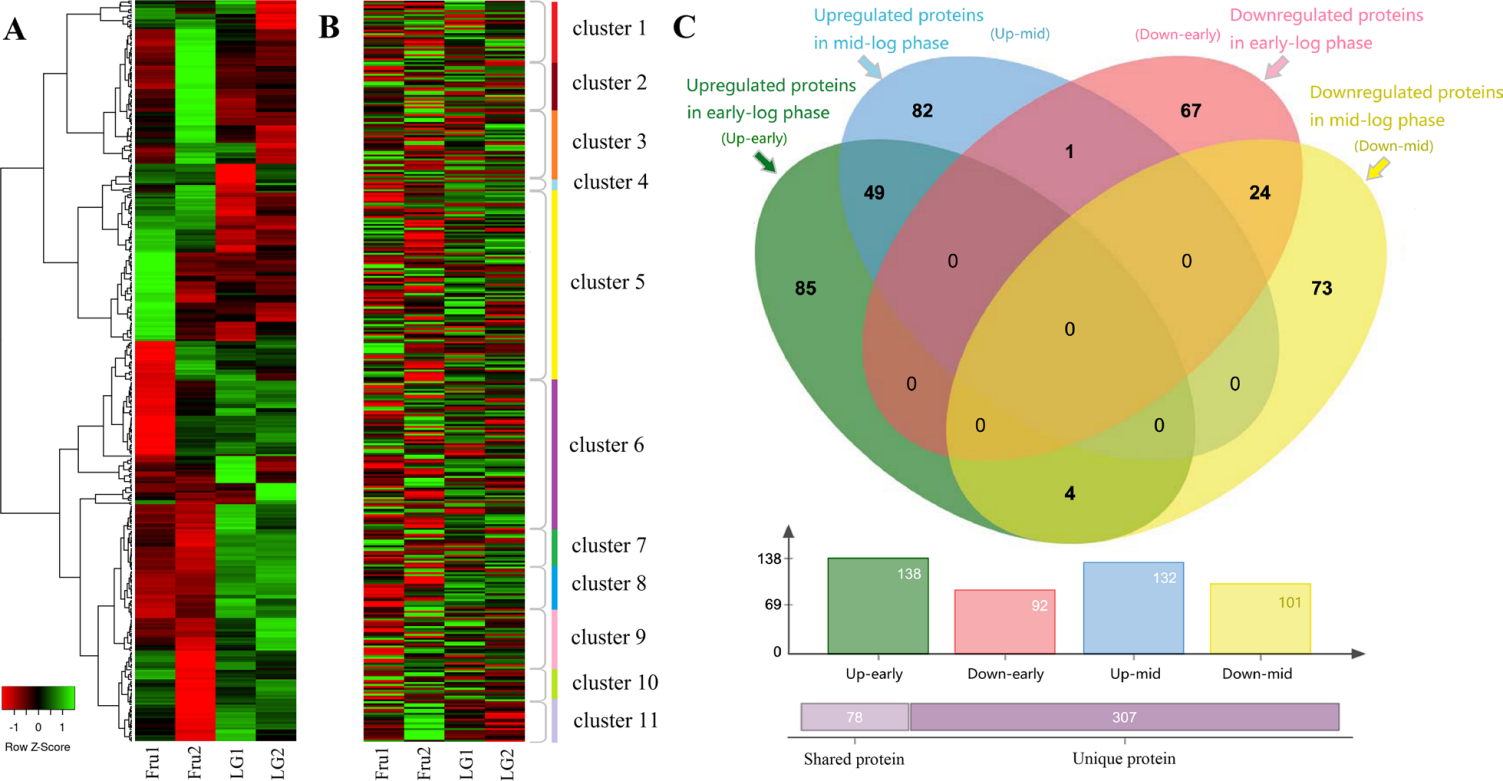
Comparison of total identified proteins and differentially expressed proteins (DEPs) of fructose-feeding and levoglucosan-feeding cells. *E. coli* LGE2 cells were cultured at 37 °C and 150 rpm in levoglucosan- or fructose-based M9 minimal media. Fru1 and LG1 denote the cells fed with fructose and levoglucosan, respectively, were harvested at the early-log growth phase; while Fru2 and LG2 denote the cells fed with fructose and levoglucosan, respectively, were harvested at mid-log growth phase. **A** Heatmap analysis of the total identified proteins in all the samples, and dendrogram shows the relationship of samples in protein expression. **B** Heatmap analysis coupled with COG categories of the total identified proteins in all the samples. Clusters 1 represents cell wall/membrane/envelope biogenesis and cell motility; Clusters 2 represents amino acid transport and metabolism; Clusters 3 represents energy production and conversion; Clusters 4 represents nucleotide transport and metabolism; Clusters 5 represents carbohydrate transport and metabolism; Clusters 6 represents DNA replication, recombination, repair, transcription, and RNA translation, ribosomal structure and biogenesis; Clusters 7 represents lipid transport and metabolism; Clusters 8 represents inorganic ion transport and metabolism; Clusters 9 represents defense and signal transduction mechanisms; Clusters 10 represents secondary metabolites biosynthesis, transport and catabolism; Clusters 11 represents poorly characterized proteins. **C** Number of unique and shared DEPs in levoglucosan-feeding cells relative to fructose-feeding cells at both the early- and mid-log phases

Our DIA (data-independent acquisition)-based quantitative proteomics results identified 2749 proteins present in all the samples (Additional file 1). Expression levels of these proteins were compared globally and shown as heatmap (Fig. 1A), which indicates that protein expression in the four samples varied significantly. All quantifiable proteins with twofolds change expression levels and Bonferroni-adjusted *p* value less than 0.05 were defined as DEPs. The DEPs with fold change ratio ≥ 2 were considered upregulated proteins (Table 1), whereas ≤ 0.5 were considered downregulated proteins (Table 2). The clustering of the DEPs according to the COG (Cluster of Orthologous Groups) categorization is shown in Fig. 1B. Also, Venn diagrams showing the number of shared and unique upregulated/downregulated proteins in all cases are presented in Fig. 1C, which shows 49 upregulated and 24 downregulated proteins were shared by both the early- and mid-log phases. Category distribution and enrichment clustering of the DEPs based on Gene Ontology (GO) analysis were shown in Additional file 2: Fig. S1. Pathway distribution and enrichment clustering of the DEPs based on Kyoto Encyclopedia of Genes and Genomes (KEGG) analysis were exhibited in Additional file 2: Fig. S2. The detailed GO and KEGG analysis were presented in Additional file 2: Text S1. From the COG, GO, and KEGG analysis, it is evident that the DEPs are mainly membrane proteins related to carbohydrate transport, localization, and metabolism, consistent with the fact the proteomics analysis was conducted by comparing the protein expression levels exhibited by cells fed with two different carbon substrates—levoglucosan and fructose.

**Table 1:** Up-regulated proteins at both early- and mid-log phases of levoglucosan utilization relative to fructose utilization

Differentially expressed proteins (DEPs)	Accession No.	Gene name	Log_2_ FC	
			Early log phase	Mid log phase
Cell wall/membrane/envelope biogenesis and cell motility-related proteins				
Maltoporin	A0A140NEY2	*lamB*	2.71	1.33
Efflux transporter, RND family, MFP subunit	A0A140NCH5	*cusB*	1.85	2.47
Flagellar basal-body rod protein FlgG	A0A140NB02	*flgG*	1.93	2.01
Energy production and conversion‑related proteins				
*N*-Succinylglutamate 5-semialdehyde dehydrogenase	A0A140N931	*astD*	2.64	4.35
Radical SAM domain protein	A0A140N975	*ydeM*	2.14	1.05
Iron-containing alcohol dehydrogenase	A0A140N5B5	*yqhD*	3.08	2.71
Uncharacterized protein	A0A140N897	*glcF*	1.93	1.42
Amino acid transport and metabolism‑related proteins				
Extracellular solute-binding protein family 1	A0A140NBZ6	*ydcS*	1.49	1.62
Extracellular solute-binding protein family 3	A0A140N2V7	*yhdW*	2.19	1.89
Tryptophanase	A0A140NGF8	*tnaA*	2.37	2.92
Nucleotide transport and metabolism‑related proteins				
Protein NrdI	A0A140N8P5	*nrdI*	2.26	1.64
Carbohydrate transport and metabolism‑related proteins				
Maltodextrin-binding protein	A0A140NCD0	*malE*	2.34	3.34
ABC-type sugar transport system periplasmic component	A0A140N593	*yphF*	1.57	2.21
ABC transporter related	A0A140NAC2	*mglA*	1.44	1.21
Extracellular solute-binding protein family 1	A0A140N4W8	*ugpB*	2.16	1.77
Phosphoenolpyruvate synthase	A0A140NB77	*ppsA*	2.25	1.87
d-Xylose ABC transporter, periplasmic substrate-binding protein	A0A140N4K7	*xylF*	2.71	3.18
Gluconokinase	A0A140N6M3	*gntK*	1.14	1.50
Gluconokinase	A0A140NGU6	*idnK*	1.25	1.27
Xylose isomerase	A0A140N6S9	*xylA*	2.63	2.69
Periplasmic binding protein/LacI transcriptional regulator	A0A140NEX7	*ytfQ*	2.14	2.49
Alpha,alpha-phosphotrehalase	A0A140NGD0	*treC*	2.06	3.50
sn-glycerol-3-phosphate import ATP-binding protein UgpC	A0A140N2F0	*ugpC*	1.47	1.22
Gluconate transporter	A0A140N385	*gntT*	1.44	1.03
Metabolite/H^+^ symporter, major facilitator superfamily (MFS)	A0A140N8T9	*kgtP*	2.47	2.74
Glyoxylate carboligase	A0A140NEP9	*gcl*	3.83	3.61
Levoglucosan kinase	–	*lgk*	2.87	3.39
Lipid transport and metabolism‑related proteins				
Acetyl-coenzyme A synthetase	A0A140NGU5	*acs*	2.63	2.57
3-Ketoacyl-CoA thiolase	A0A140NDQ6	*fadA*	1.66	1.81
Long-chain-fatty-acid–CoA ligase	A0A140N8F0	*fadD*	1.99	1.33
DNA replication, recombination, repair, transcription, and RNA translation‑related proteins				
Transcriptional regulator, LacI family	A0A140N9Y3	*galS*	1.63	1.91
Transcriptional regulator, IclR family	A0A140NF20	*mhpR*	1.36	1.40
Transcriptional regulator, AraC family	A0A140NDL9	*ykgD*	3.51	1.33
30S ribosomal subunit S22	A0A140NCE1	*sra*	1.26	1.11
Regulator of sigma D	A0A140SS61	*rsd*	2.28	1.17
Transcriptional regulator, LacI family	A0A140N2K5	*ybl162*	1.16	1.55
Inorganic ion transport and metabolism‑related proteins				
Periplasmic copper-binding protein	A0A140NEZ0	*cusF*	1.96	2.55
Sulfatase	A0A140N782	*ydeN*	1.20	2.23
Heavy metal efflux pump, CzcA family	A0A140NCW8	*cusA*	2.23	1.38
Cation/acetate symporter ActP	A0A140SS45	*actP*	3.12	2.73
Defense and signal transduction mechanisms‑related proteins				
Sulfatase	A0A140NB15	*ybiP*	3.40	1.84
Protein tyrosine phosphatase	A0A140NB74	*yccY*	1.15	1.69
Secondary metabolites biosynthesis, transport and catabolism‑related proteins				
Mammalian cell entry related domain protein	A0A140N6C3	*yebT*	1.70	1.16
Poorly characterized proteins				
Maltose operon periplasmic	A0A140NFH4	*malM*	1.50	2.67
DUF1338 domain-containing protein	A0A140N7F7	*ydcJ*	1.59	1.41
Anti-adapter protein IraP	A0A140NB68	*iraP*	1.85	1.02
PEBP family protein	A0A140NF09	*ybcL*	1.01	1.34

**Table 2:** Down-regulated proteins at both early- and mid-log phases of levoglucosan utilization relative to fructose utilization

Differentially expressed proteins (DEPs)	Accession No.	Gene name	Log_2_ FC	
			Early log phase	Mid log phase
Energy production and conversion‑related proteins				
FdrA family protein	A0A140NAI7	*yahF*	− 2.42	− 2.17
Aldehyde-alcohol dehydrogenase	A0A140NCE4	*adhE*	− 1.82	− 1.52
Molybdopterin dehydrogenase FAD-binding	A0A140N5N6	*ygeT*	− 3.02	− 1.07
Cytochrome o ubiquinol oxidase, subunit III	A0A140NC92	*cyoC*	− 2.03	− 1.29
Molybdopterin oxidoreductase Fe_4_S_4_ region	A0A140NE68	*fdhF*	− 1.20	− 2.82
Hydrogenase (NiFe) small subunit HydA	A0A140NDP4	*hyaA*	− 2.10	− 1.06
Nickel-dependent hydrogenase large subunit	A0A140NB83	*hyaB*	− 1.05	− 1.20
Amino acid transport and metabolism‑related proteins				
Inner-membrane translocator	A0A140N716	*livH*	− 1.03	− 2.99
T-protein	A0A140N544	*tyrA*	− 1.66	− 2.29
Carbohydrate transport and metabolism‑related proteins				
Fructose-bisphosphate aldolase	A0A140N821	*fba*	− 1.56	− 1.27
d-Erythrose-4-phosphate dehydrogenase	A0A140N827	*epd*	− 1.97	− 1.35
Ribulose-phosphate 3-epimerase	A0A140SS41	*alsE*	− 1.23	− 4.07
PTS system, fructose subfamily, IIC subunit	A0A140N8J0	*fruA*	− 3.24	− 4.43
Phosphofructokinase	A0A140N679	*fruK*	− 3.67	− 3.81
PTS system fructose-specific EIIA component	A0A140N9Z8	*fruB*	− 4.11	− 3.59
Replication, recombination and repair‑related proteins				
Integration host factor subunit beta	A0A140NDV2	*ihfB*	− 1.51	− 1.22
Poorly characterized proteins				
5′-Deoxynucleotidase YfbR	A0A140N9N4	*yfbR*	− 5.22	− 4.74
Protein ViaA	A0A140NFS7	*viaA*	− 1.25	− 1.12
ATPase RavA	A0A140NI88	*ravA*	− 1.43	− 1.18
Phage minor tail protein G	A0A140NE00	*ECBD_2862*	− 1.22	− 3.06
Protein YcfR	A0A140N4R7	*ycfR*	− 2.54	− 2.45
Type VI secretion system effector, Hcp1 family	A0A140N758	*yhhZ*	− 1.27	− 5.48

### Several carbohydrate transport-related DEPs were upregulated in response to levoglucosan uptake relative to fructose uptake

*Escherichia coli* has an outer membrane and an inner cytoplasmic membrane, and the space between them is periplasm. The outer membrane protein LamB (A0A140NEY2), as a sugar porin protein that specifically facilitates the passive diffusion of many carbohydrates, including trehalose, lactose, sucrose, maltose, maltodextrins and glucose, and other non-specific ion/solutes across the outer membrane [23, 24], was 2.7-fold upregulated at the early-log phase and 2.0-fold at the mid-log phase when induced by levoglucosan, indicating the role of LamB in levoglucosan transport from the ambient medium to the periplasm.

**Fig. 2:**
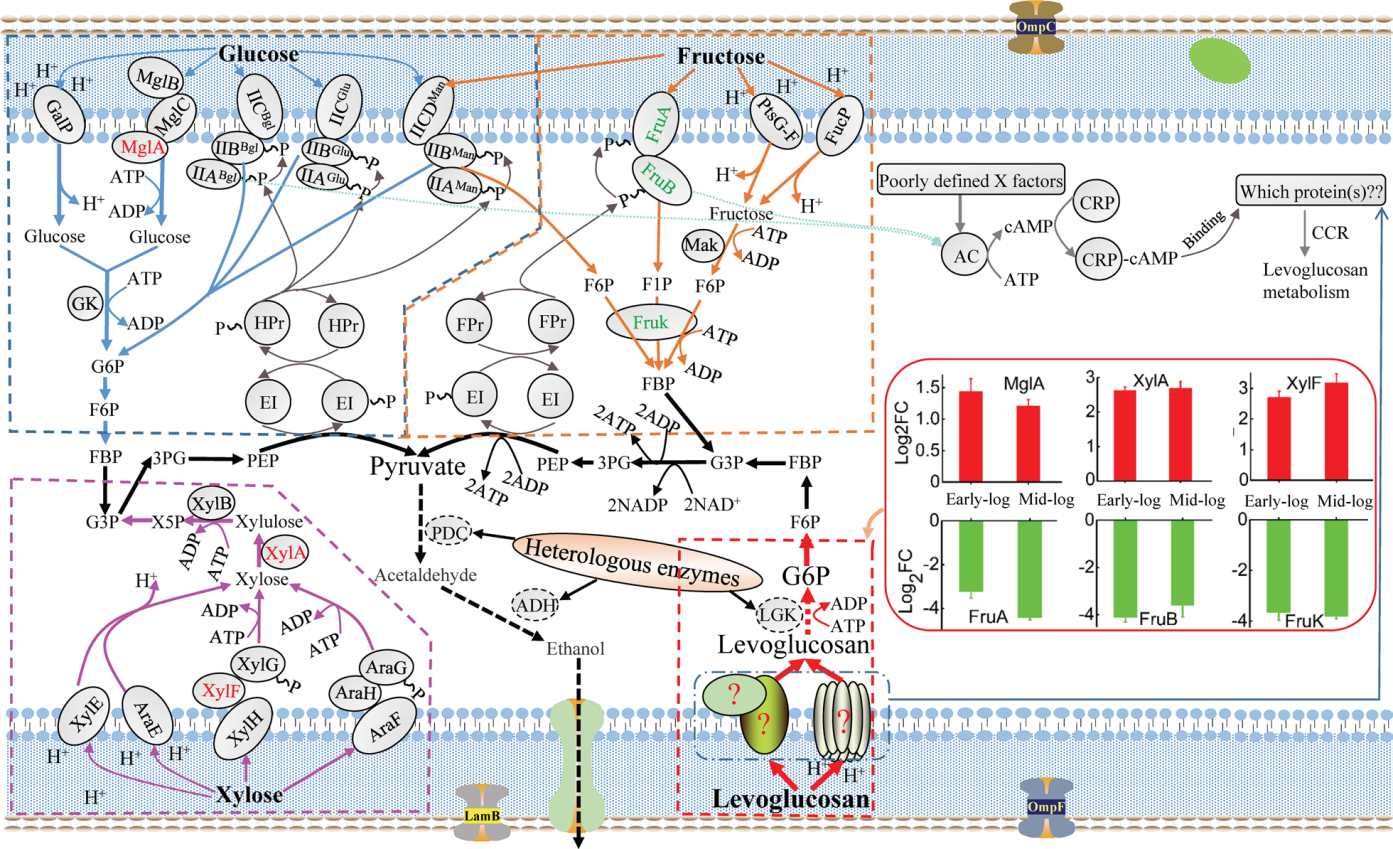
The (potential) transport components and transport mechanisms of different sugars in *E. coli*. The enzymes in red color were upregulated during levoglucosan utilization relative to fructose utilization, while those in green color were downregulated

Upon entering the periplasm, sugars are further transported into the cytoplasm and phosphorylated by different mechanisms (Fig. 2). The native sugar transporters of *E. coli* mainly include the phosphoenolpyruvate (PEP)-dependent carbohydrate phosphotransferase system (PTS), the ATP-binding cassette (ABC) transporter, and the major facilitator superfamily (MFS) [25]. Among the differentially expressed transporter proteins, only fructose-specific FruA (A0A140N8J0) and FruB (A0A140N9Z8) that can transport and phosphorylate fructose [26] are PEP-PTS proteins, and both were downregulated by about 9.4- and 17.3-fold at the early-log phase and 21.6- and 12.0-fold at the mid-log phase, respectively. By FruA and FruB, fructose can be transported into *E. coli* and phosphorylated to fructose 1-phosphate (F1P) or fructose 6-phosphate (F6P) [27], which are further phosphorylated to fructose 1,6-bisphosphate (FBP) by phosphofructokinase (FruK) and metabolized by *E. coli* (Fig. 2). Our result that proteins FruA, FruB, and FruK (FruK is discussed in the sections below) were all downregulated in levoglucosan-based media compared to the fructose-based media at both early- and mid-log phases, is consistent with the fact that the *fru* operon sequentially containing the *fruB*, *fruK*, and *fruA* genes is induced by fructose.

The ABC transporters such as XylF (A0A140N4K7), MalE (A0A140NCD0), UgpB (A0A140N4W8), UgpC (A0A140N2F0), YtfQ (A0A140NEX7), YphF (A0A140N593), and MglA (A0A140NAC2) were upregulated at both the early- and mid-log phases of levoglucosan consumption compared to those of fructose consumption (Table 1). The d-xylose ABC transporter substrate-binding protein XylF as a periplasmic binding protein is involved in the ATP-dependent high-affinity xylose uptake system [28], and in this study, it was upregulated by about 6.5- and 9.1-fold at early- and mid-log phases, respectively. MalE, as the essential periplasmic binding protein component of the maltose MalKFGE ABC transporter, is responsible for the maltose uptake and was up-regulated by about 5.1- and 10.1-fold, respectively. The glycerol 3-phosphate (G3P) ABC transporters UgpB and UgpC, galactofuranose ABC transporter YtfQ, putative sugar ABC transporter substrate-binding protein YphF, and d-galactose/d-galactoside ABC transporter MglA were upregulated by about 4.5, 2.8, 4.4, 3.0, and 2.7-fold at the early-log phase, and by about 3.4, 2.3, 5.6, 4.6, and 2.3-fold at the mid-log phase, respectively. The MFS proteins like KgtP (A0A140N8T9), GntT (A0A140N385), and ActP (A0A140SS45) were also upregulated at both phases. During levoglucosan consumption at the early- and mid-log phases, the proton-driven α-ketoglutarate transporter KgtP consisting of many transmembrane spanning segments and sugar transport domains was upregulated by about 5.5- and 6.7-fold, respectively; gluconate transporter GntT involved in the gluconate uptake system driven via d-gluconate/proton symport was upregulated by about 2.7- and 2.0-fold, respectively; ActP, an acetate/glycolate permease in the solute: sodium symporter family, was also upregulated by respective 8.7- and 6.6-fold. All these upregulated transporters might be related to levoglucosan uptake, because their expression levels were higher in the presence of levoglucosan, and it is known that most transporters can transport not only one substrate.

In the LGK-catalyzing pathway, levoglucosan is phosphorylated but not transported by the non-PTS kinase LGK [3], which was highly expressed in response to levoglucosan metabolism (more proteins involved in carbohydrate metabolism and energy production were shown in Additional file 2: Text S2); therefore, it could be assumed that, some unknown non-PTS transporters coupled with a proton motive force (H^+^) or a direct energy drive (ATP) (Fig. 2), might be involved in the transport of levoglucosan. Interestingly, our results experimentally indicate that all the levoglucosan-induced transporters were non-PTS ABC and MFS transporters rather than PTS transporters. Although the identified ABC and MFS transporters induced by levoglucosan have been known to be involved in the transport of other non-levoglucosan sugars (described above), it is believed that these highly induced transporters might be responsible for the transport of levoglucosan into the cell cytoplasm. In fact, many sugar transporters can transport more than one substrate. For example, apart from the PEP-PTS-dependent glucose transporter PtsG; the non-PTS-dependent galactose: H^+^ symporter GalP, non-PTS-dependent galactose import-related MglABC, and PEP-PTS-dependent β-glucoside-specific transporter Bgl mainly responsible for the transport of galactose, galactoside, and β-glucoside, respectively, can also transport glucose, and the PEP-PTS-dependent mannose-transport carriers ManXYZ can transport both glucose and fructose (Fig. 2). Moreover, fucose transporter FucP, and arabinose transporters AraE and AraFGH can also transport fructose and xylose, respectively. In addition, in *Aspergillus nidulans*, HxtB, previously considered as a glucose transporter, has been recently proved to be a major xylose transporter [29], and the monosaccharide transporter XtrD turned out to have a high affinity for xylose in *A. nidulans* [30]. Consequently, the levoglucosan-inducing transporters MglA, XylF, MalE, UgpB, UgpC, YtfQ, YphF, KgtP, GntT, and ActP supposed to be related to levoglucosan transport could provide a database for further screening of levoglucosan transporters, which could contribute to the development of robust levoglucosan-utilizing strains.

### Most DEPs related to transcription and regulation were upregulated in response to levoglucosan metabolism relative to fructose metabolism

CCR is another bottleneck that cannot be ignored in the process of levoglucosan uptake and metabolism. The preferential utilization of the most available sugar is an adaptation of bacteria to survive in a competitive environment. However, CCR inhibits the efficient production of bioproducts in industrial fermentation by reducing the conversion efficiency of preferred secondary sugars and increasing the whole fermentation time. In *E. coli*, there are two dominant transcriptional regulation mechanisms involved in the CCR of carbon metabolism; one is through the *crp*-encoded cyclic AMP receptor protein (Crp) that regulates the initiation of carbon metabolism, and the other by the *cra (fruR)*-encoded catabolite repressor/activator (Cra) protein that frequently regulates carbon flux through the dominant metabolic pathways [31]. In the CCR of carbon sources, PTS forms part of the regulation network, while global and operon-specific regulations also control the CCR (Fig. 2).

In the current study, DEPs related to transcription and regulation like GalS (A0A140N9Y3), MhpR (A0A140NF20), YkgD (A0A140NDL9), Rsd (A0A140SS61), Ybl162 (A0A140N2K5), MalM (A0A140NFH4), and IraP (A0A140NB68) were all induced by levoglucosan at both phases (Tables 1 and 2). GalS, as a CRP-dependent DNA-binding transcription factor that represses transcription of the operons involved in transport and catabolism of d-galactose and can be stimulated by the addition of d-fucose [32], was upregulated by about 3.1- and 3.8-fold at respective growth phase. MhpR, as a 3-(3-hydroxyphenyl) propionic acid-dependent activator of the Pa promoter that controls the expression of the *mhp* catabolic gene and is essential for the binding of CRP [33], was upregulated by about 2.5- and 2.6-fold. DNA-binding and redox-regulated transcriptional activator YkgD that can be induced by oxidation of its highly conserved cysteine residues [34] was upregulated by about 11.4- and 2.5-fold, the highest average fold change value we observed among the transcription and regulation related DEPs. Regulator of σ70 Rsd functioning as a link between PTS-dependent carbon source utilization and the stringent response phosphocarrier protein HPr, which is one of two sugar-non-specific protein constituents of the PEP-PTS sugar [35], was upregulated by about 4.9- and 2.3-fold. Ybl162 as a LacI family transcriptional regulator predicted by automated computational analysis was upregulated by about 2.2- and 2.9-fold. MalM as the last gene of the *malK-lamB-malM* operon and part of the maltose regulon was upregulated by about 2.8- and 6.4-fold, consistent with the upregulation pattern of LamB. Anti-adapter protein IraP that can increase the stability of the sigma stress factor RpoS by inhibiting RpoS proteolysis was upregulated by about 3.6- and 2.0-fold. The upregulation pattern of these proteins suggested their possible roles in the regulation of levoglucosan metabolism; especially, YkgD that was highly induced and GalS and MhpR that are CRP-related proteins might directly contribute to the CCR of levoglucosan.

CRP is a global regulator and exhibits pleiotropic phenotypes by forming a complex with cAMP, and then the CRP–cAMP complex-mediated CCR makes *E. coli* cells preferentially metabolize glucose over fructose over xylose [36] and levoglucosan [20]. When the catabolite repressor/activator gene *cra* that negatively regulates the *fru* operon is deleted in *E. coli*, the mutant strain without repression of *fru* operon (FruAB and FruK) by glucose can co-utilize glucose and fructose [37]. The xylose-specific operons (*xylE*, *xylFGHR,* and *xylAB*) are under the regulation of XylR and cAMP-CRP-system regulator, and are also repressed by Mlc-regulated genes, including *ptsG* and *manXYZ* [38]. When fed with mixed sugars of glucose, arabinose, and xylose, *E. coli* cells first consume glucose, then arabinose, and finally xylose. Deleting gene *ptsG* makes *E. coli* co-utilize arabinose with glucose, although xylose utilization remains repressed by arabinose. Further attempts to replace the native *crp* gene with a cAMP-independent mutant without CCR can facilitate the simultaneous utilization of glucose, arabinose, and xylose [39]. A cis-acting DNA element known as the catabolite responsive element (*cre*) located within the open reading frame of *xylA* contributes to the CCR of xylose; accordingly, the strain with an inactivated *cre* site in *xylA* could consume fructose and xylose simultaneously [40], but this strain still exhibited diauxic growth on glucose and xylose. Therefore, CCR is a phenomenon resulting from many complex factors.

For the CCR involved in levoglucosan consumption, when glucose and fructose are absent in the culture media, adenylate cyclase (AC) can be activated by the phospho-form of glucose-specific PTS enzyme EIIA^Glc^, β-glucoside-specific PTS enzyme EIIA^Bgl^, and fructose-specific PTS enzyme EII^Fru^ [27], thus improving the cellular cAMP level; then the formed cAMP–Crp complex will activate the transmembrane transporters responsible for levoglucosan uptake (Fig. 2). In addition, AC activity can also be regulated by a GTP-binding elongation factor Tu [41] and several uncharacterized regulatory factors (poorly defined X factors) that are required for the effective coupling of PTS proteins to AC [42–44]. Therefore, these poorly defined X factors might also contribute to the uptake of levoglucosan. In combination with our proteomics results, it is anticipated that the transcription and regulation-coupled proteins related to CCR like YkgD, GalS, and MhpR (Table 1), which might be the X factors, could improve our understanding of the biological regulation processes to relieve the CCR of levoglucosan utilization by further genetic manipulations.

### RT-PCR results validated the reliability of DIA-based proteomics results

**Fig. 3:**
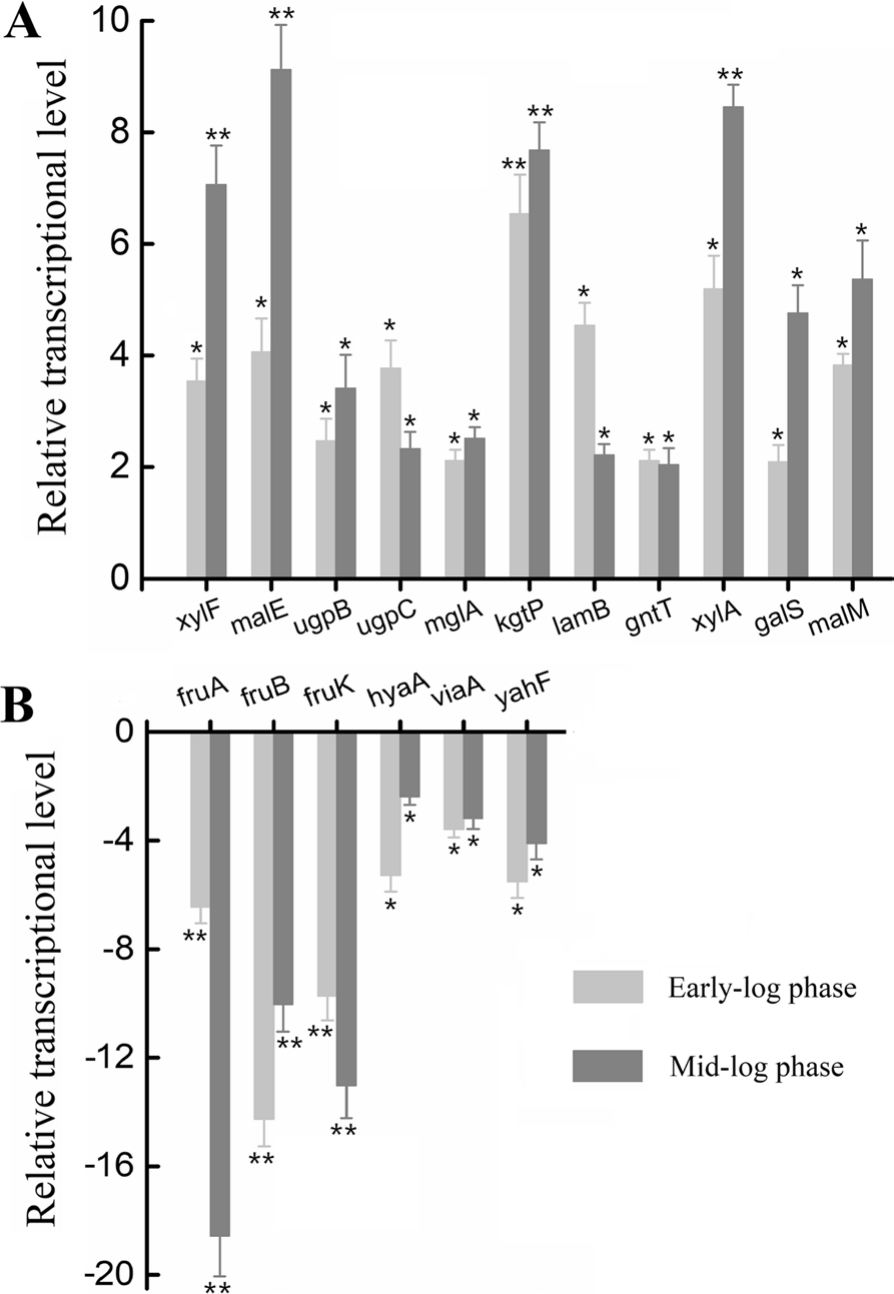
The relative transcriptional levels of several randomly-selected genes during levoglucosan utilization relative to fructose utilization. *E. coli* LGE2 cells were cultured at 37 °C and 150 rpm in levoglucosan- and fructose-based M9 minimal media, and then harvested at both the early- and mid-log phases. **A** The upregulated mRNAs. **B** The downregulated mRNAs. The light grey column denotes the mRNA was sampled at early-log phase, while the dark grey column denotes that at mid-log phase

We also measured the transcriptional levels of 16 randomly selected DEPs described above at both early- and mid-log phases of levoglucosan metabolism using quantitative RT-PCR to validate the protein expression data obtained by DIA-based quantitative proteomics (Fig. 3). In all, the quantitative RT-PCR results for *xylF*, *malE*, *ugpB*, *ugpC*, *mglA*, *kgtP*, *lamB*, *gntT*, *xylA*, *galS*, *malM*, *fruA*, *fruB*, *fruK*, *hyaA*, *viaA*, and *yahF* are consistent with the relative quantitative protein expression results. The genes *xylF*, *malE*, *ugpB*, *ugpC*, *mglA*, *kgtP*, *lamB*, *gntT*, *xylA*, *galS*, and *malM* were all transcriptionally upregulated in levoglucosan-feeding cells compared to fructose-feeding cells, with the same expression direction to the protein expression results (Fig. 3). Of the upregulated genes, *xylF*, *malE*, *kgtP*, and *xylA* exhibited significantly higher fold changes in the transcript level than others, especially at the mid-log phase (*p* < 0.01). Moreover, the transcriptional levels of genes *fruA*, *fruB*, *fruK*, *hyaA*, *viaA*, and *yahF* were downregulated in levoglucosan-feeding cells (Fig. 3). Of these downregulated genes, the changes of *fruA*, *fruB*, and *fruK* in the transcript level were significant (*p* < 0.01), with a high fold-change ratio. Consequently, the quantitative RT-PCR results evidenced and strengthened the reliability of the relative quantitative protein expression results determined using DIA-based proteomics.

### Levoglucosan consumption was decreased by the deletion of genes *kgtP* and *xylF* while resumed by their complementation

Taking the proteomics and RT-PCR results together (mainly according to the fold change values of the protein and mRNA expression level), we proceeded to determine whether ABC transporter XylF and MFS transporter KgtP were related to levoglucosan uptake and metabolism, although it has been known that XylF is a ATP-dependent ABC xylose transporter [28] and KgtP is a proton-driven α-ketoglutarate transporter [45]. Based on the pCasPA/pACRISPR genome editing system, numerous colonies, in which the *xylF* or *kgtP* gene should be deleted, were successfully grown on the antibiotics screening plates. Due to the presence of the sucrose-inducing suicide gene SacB within the pCasPA and pACRISPR plasmids, both plasmids can be eliminated by sucrose selection, and then the randomly selected plasmid-eliminated colonies were re-verified by antibiotics (tetracycline and ampicillin) pressure and PCR (Additional file 2: Fig. S4) before sequencing. Our results showed that the genome editing system has higher efficiency in creating mutations than traditional methods, as 100% (10/10) of the randomly selected colonies were gene-deleted strains.

**Fig. 4:**
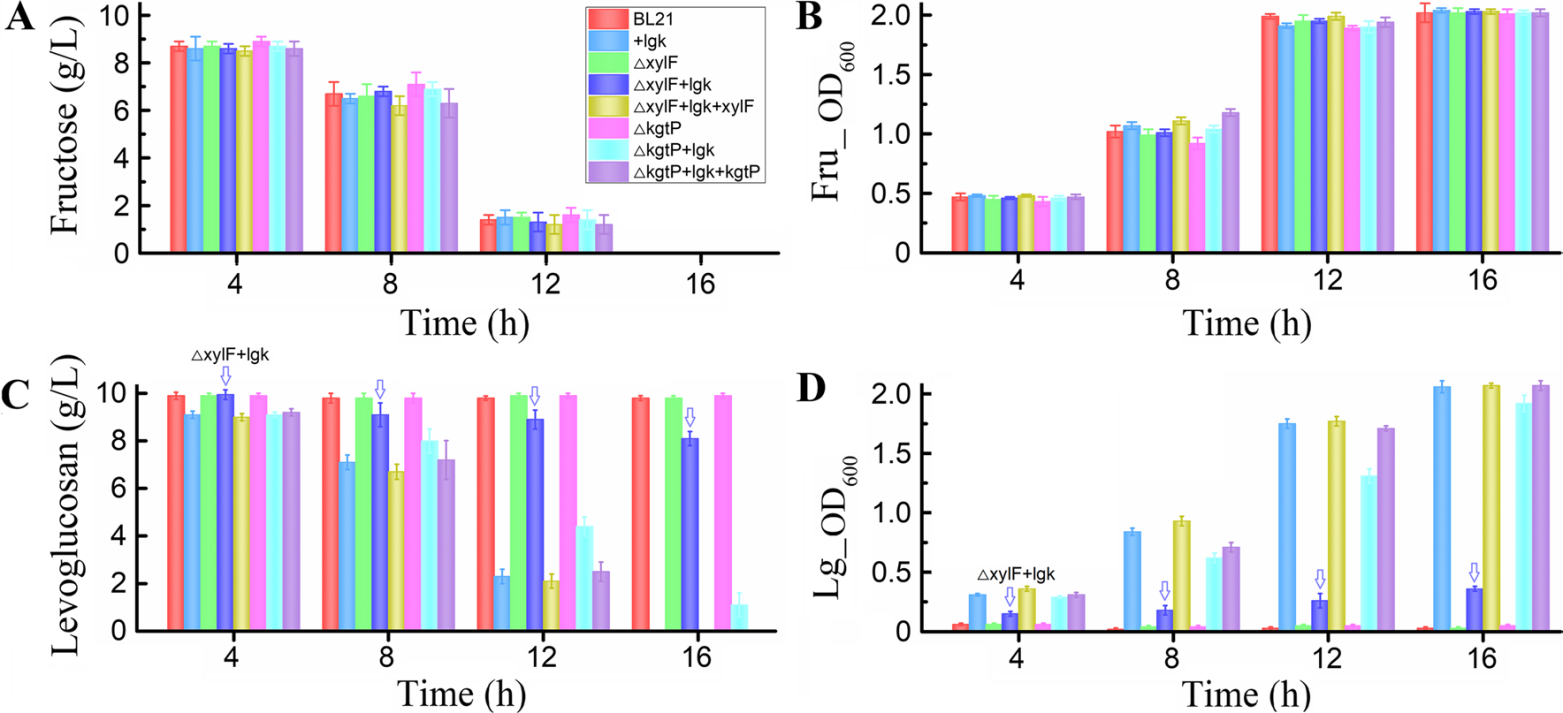
The time-course profiles of cell growth and sugar utilization of engineering and non-engineering *E. coli*. *E. coli* BL21 (DE3), *E. coli* (pET-*lgk*), *E. coli* Δ*xylF*, *E. coli* Δ*kgtP*, *E. coli* Δ*xylF* + *lgk*, *E. coli* Δ*kgtP* + *lgk*, *E. coli* Δ*xylF* + *lgk* + *xylF*, and *E. coli* Δ*kgtP* + *lgk* + *kgtP* were cultured at 37 °C and 150 rpm for 24 h in levoglucosan- and fructose-based M9 minimal media, respectively. *E. coli* (pET-*lgk*) is abbreviated to *E. coli* + *lgk*. **A** Fructose consumption and **B** Cell density (OD_600_) in fructose-feeding media. **C** Levoglucosan consumption and **D** cell density (OD_600_) in levoglucosan-feeding media. Downward arrows labeled in the figures highlighted the levoglucosan consumption and cell density exhibited by *E. coli* Δ*xylF* + *lgk*

Furthermore, the utilization of different sugar substrates—levoglucosan and fructose (as a control) were investigated to determine the effects of *xylF* or *kgtP* deletion on levoglucosan as well as fructose utilization. The *xylF*-deleted strain *E. coli* Δ*xylF*, *xylF*-deleted and plasmid-borne *lgk* (levoglucosan kinase gene)-introduced strain *E. coli* Δ*xylF* + *lgk*, and *xylF*-complemented and *lgk*-introduced strain *E. coli* Δ*xylF* + *lgk* + *xylF* showed similar cell growth profiles and fructose-utilizing abilities to the parent strain *E. coli* BL21 and the *lgk*-introduced strain *E. coli* + *lgk* (Fig. 4A, B), implying that deletion of *xylF* had no apparent effect on the fructose utilization of the *E. coli* strain. In parallel, the *kgtP*-deleted and complemented *E. coli* strains Δ*kgtP,* Δ*kgtP* + *lgk*, and Δ*kgtP* + *lgk* + *kgtP* also showed no apparent discrepancies in cell growth and fructose utilization (Fig. 4A, B). However, in respect to the levoglucosan utilization, the levoglucosan consumption and cell growth of *xylF*/*kgtP*-deleted and *lgk*-introduced strains *E. coli* Δ*xylF* + *lgk* and Δ*kgtP* + *lgk* were both slower than that of the control strain *E. coli* + *lgk*; especially, the *xylF*-deleted strain *E. coli* Δ*xylF* + *lgk* showed a remarkably poor ability of levoglucosan consumption and cell growth (Fig. 4C, D). After a 16-h incubation, *E. coli* + *lgk* could consume all the levoglucosan and reach a maximum cell density (OD_600_) of 2.07 while *E. coli* Δ*xylF* + *lgk* and Δ*kgtP* + *lgk* could not; deletion of *xylF* and *kgtP* resulted in a levoglucosan residue of about 8.1 and 1.0 g/L, respectively (Fig. 4C, D). At the next sampling point (20 h), all the levoglucosan was utilized by *E. coli* Δ*kgtP* + *lgk*; however, *E. coli* Δ*xylF* + *lgk* still could not efficiently consume the levoglucosan, with about 6.9 g/L of levoglucosan remaining in the media. Furthermore, complementation of *xylF* and *kgtP* restored the destroyed genes and rendered the levoglucosan consumption and cell growth rates comparable to that of the control strain *E. coli* + *lgk* (Fig. 4C, D, and Table 3). These results showed that levoglucosan utilization was delayed by the separate deletion of both genes, indicating that both XylF and KgtP are related to the transport and metabolism of levoglucosan. However, XylF was more likely to be an effective levoglucosan transporter than KgtP, as deletion of *xylF* affected the levoglucosan consumption rate and growth of *E. coli* more significantly than deletion of *kgtP* (*p* < 0.01) (Table 3).

**Table 3:** The maximal specific growth rate *μ*_max_ (h^−1^) of the gene-deleted/complemented *E. coli* strains grown in fructose- and levoglucosan-based minimal media

Substrate	BL21	+ *lgk*	Δ*xylF*	Δ*xylF* + *lgk*	Δ*xylF* + *xylF* + *lgk*	Δ*kgtP*	Δ*kgtP* + *lgk*	Δ*kgtP* + *kgtP* + *lgk*
Fructose	0.56^(0.01)^	0.55^(0.02)^	0.52^(0.03)^	0.52^(0.01)^	0.54^(0.02)^	0.50^(0.04)^	0.51^(0.03)^	0.53^(0.04)^
Levoglucosan	0	0.51^(0.01)^	0	0.09^(0.03)^	0.56^(0.04)^	0	0.39^(0.03)^	0.50^(0.02)^

All the values in the table for this research are average of triplicate samples. The superscript value in the parentheses is the standard deviation

Although we, for the first time, identified that XylF and KgtP, especially XylF, were related to the microbial levoglucosan utilization; currently, characterization of the enzymatic parameters of XylF and KgtP for levoglucosan uptake is still problematic because no method related to levoglucosan uptake has been developed so far. However, referring to the characterization of glucose/xylose transporters [46, 47], the isotope labeling method for characterization of levoglucosan transporter(s) would be a promising solution in the future study. Moreover, deletion of the two genes did not result in complete interruption of levoglucosan utilization (Fig. 4), implying that other unknown transporters for levoglucosan transport also exist. For the identification of specific transporter of levoglucosan, if it exists, further researches are also required.

### Molecular docking showed levoglucosan could bind to XylF with relatively high affinity

**Fig. 5:**
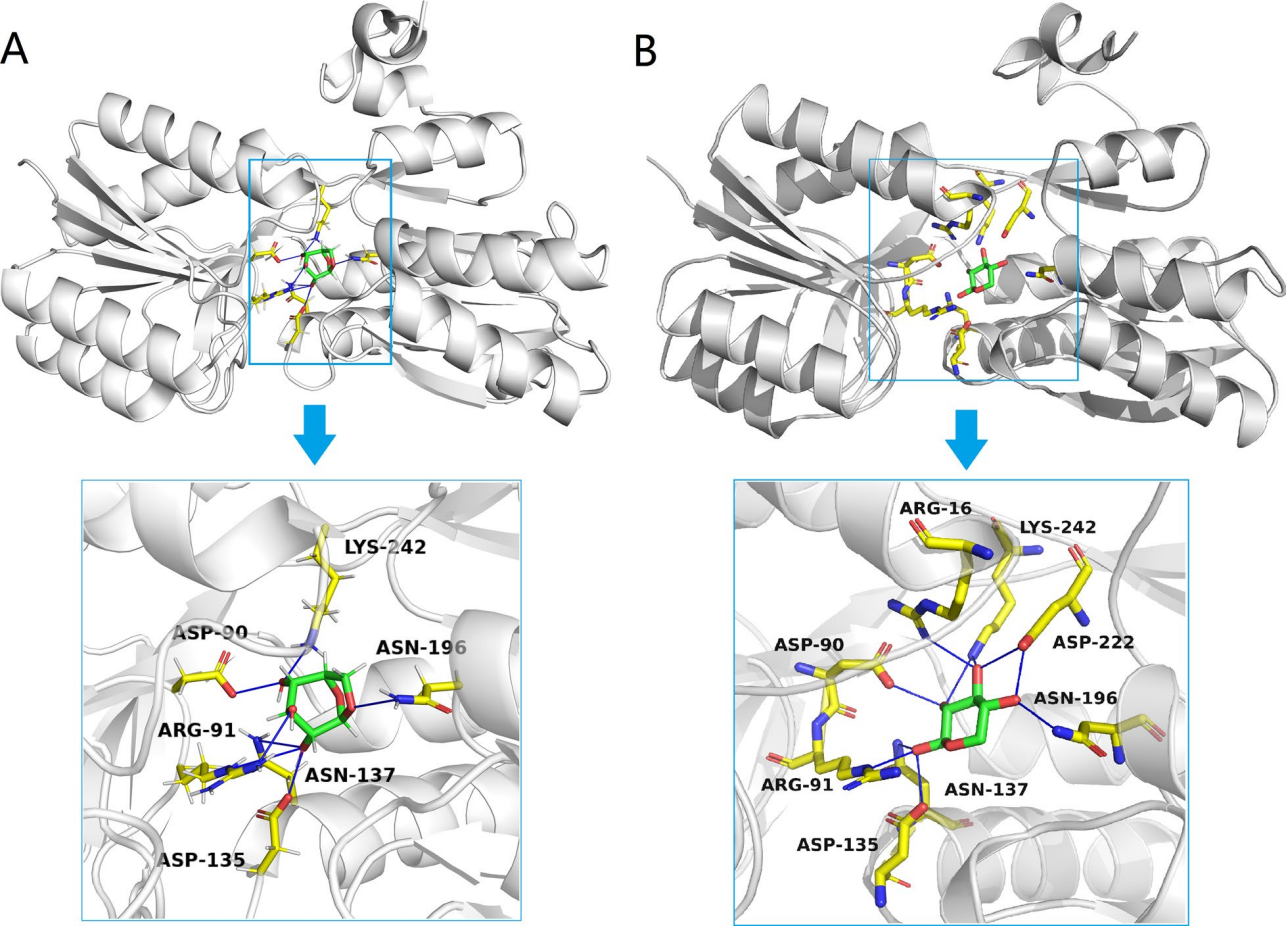
The interaction diagram of XylF with levoglucosan (**A**) and xylose (**B**). The white cartoon model is the secondary structure of XylF, the yellow stick model is the key residue of XylF, the green stick model in **A** is levoglucosan skeleton and in **B** is xylose skeleton, the red stick model is oxygen, the thick blue stick model is nitrogen, and the thin blue stick model is H-bond

Further molecular docking for modeling the binding of levoglucosan to XylF is shown in Fig. 5A. The binding energy (docking score) between XylF and levoglucosan is − 6.9 kJ/mol in the best binding conformation, suggesting levoglucosan could bind to XylF with relatively high affinity. There are six residues (Asp-90, Arg-91, Asp-135, Asn-137, Asn-196, and Lys-242) in XylF that can interact with levoglucosan by classical bidentate H-bonds (Fig. 5A and Additional file 2: Table S1). The bond length and angle of H-bond are important parameters, which represent the strength of affinity. In general, the shorter the bond length and the larger the bond angle, the stronger the bond strength. Among the H-bonds between XylF and levoglucosan, the H-bond between Asp-90 of XylF and levoglucosan had the shortest bond length (1.62 Å) and second largest bond angle (165.22°), while H-bond between Asp-135 and levoglucosan had the second shortest bond length (1.63 Å) and largest bond angle (171.72°) (Additional file 2: Table S1). In addition, Arg-91 had two H-bonds interacting with levoglucosan with respective 2.22 and 3.06 Å. This collectively implies the vital roles of Asp-90, Asp-135, and Arg-91 in the levoglucosan-binding active pocket of XylF.

Because XylF is originally found to be able to bind to xylose, we further compared the binding conformation of xylose-XylF (Fig. 5B) to that of levoglucosan-XylF (Fig. 5A). In the closed xylose-XylF structure [48], there are twelve H-bonds (Fig. 5B) between xylose and the eight residues of XylF (Arg-16, Asp-90, Arg-91, Asp-135, Asn-137, Asn-196, Asp-222, and Lys-242), five H-bonds and two interactive residues more than those of levoglucosan-XylF. Different from the levoglucosan-XylF structure, Lys-242 of xylose makes two H-bonds with XylF with the shortest length of both 2.5 Å, followed by Asp-222 forming two H-bonds with respective 2.5 and 2.6 Å, and then Asp-135 forming one H-bond with 2.6 Å [48]. In addition, Arg-91 (2.9 and 3.0 Å) and Asn-137 (2.9 and 3.1 Å) also form two H-bonds with xylose, respectively [48]. Altogether, xylose and levoglucosan can bind to XylF within similar active pocket (Fig. 5), but the binding strength is different; although the number of H-bonds between levoglucosan and XylF is much less than that between xylose and XylF, the shorter H-bond lengths between levoglucosan and XylF might exhibit slightly weaker or comparable binding strength to that of the xylose-XylF structure. Notably, it is reported that the bacterial levoglucosan dehydrogenase from *Pseudarthrobacter phenanthrenivorans* can also catalyze the oxidation of xylose [10], implying a similarity between the cellular bioconversion of levoglucosan and xylose, although the relationship between them requires further studies. Therefore, the docking results together with the above gene editing results suggest that the xylose transport-related XylF is also a levoglucosan transport-related protein that would be modified by enzymatic engineering to achieve more effective utilization of levoglucosan to develop more robust levoglucosan-converting strains.

## Conclusions

Our comparative proteomics analysis of levoglucosan and fructose utilization by engineered *E. coli* revealed many differentially expressed proteins related to carbohydrate transport and metabolism, transcription, regulation, etc. Especially, a total of ten ABC and MFS transporters were identified to be closely related to levoglucosan transport, and seven regulators were also speculated to be related to the CCR phenomenon of levoglucosan metabolism. Further gene knockout and complementation showed that transporters XylF and kgtP were both related to levoglucosan uptake and metabolism, while XylF that considerably affected the levoglucosan consumption and could bind to levoglucosan with strong H-bonds is more like a levoglucosan transporter. It is undeniable that any new screening laboratory results would need further attempts to proceed via the future study. We envision that the database generated by this study would promote a series of more profound researches devoting to the search and identification of more specific levoglucosan transporters as well as the regulation factors of CCR of levoglucosan, facilitating the development of more robust microbial strains for levoglucosan bioconversion to high value-added biofuels and chemicals.

## Materials and methods

### Microorganisms, plasmids, and culture conditions

All the strains and plasmids used in this study are listed in Table 4. The previously engineered levoglucosan-utilizing and ethanol-producing strain *E. coli* LGE2 [17] was used for proteomics analysis. *E. coli* DH5α was used for plasmid maintenance and BL21 (DE3) for plasmid transformation, gene expression, and gene knockout experiments. Plasmids pET-21a and pET-*lgk* were used for gene expression. Genome editing plasmids pCasPA and pACRISPR were purchased from the Addgene plasmid repository (Additional file 2: Figs. S5 and S6). The first-grade seed culture prepared from a single colony of *E. coli* strain was inoculated (1% v/v) into 100-mL levoglucosan-containing M9 minimal medium (7.1 g/L Na_2_HPO_4_, 3.0 g/L KH_2_PO_4_, 0.5 g/L NaCl, 1.0 g/L NH_4_Cl, 0.49 g/L MgSO_4_, 14.7 mg/L CaCl_2_, and 10.0 g/L levoglucosan). After incubating in a shaker at 37 °C and 150 rpm overnight, the second-grade seed culture was harvested at about 6 × 10^8^ cells/mL and used for subsequent experiments. Ampicillin, chloramphenicol, and tetracycline with respective final concentration of 100, 34, and 15 μg/mL were added into the media according to the antibiotic resistance of the strain used.

**Table 4:** Strains, plasmids, and primers used in this work

Strains, plasmids, or primers	Description	Source
Strains		
*E. coli* DH5α	F^−^ (80d *lac*Z M15) (*lac*ZYA-*arg*F) U169 *hsd*R17(r^−^ m^+^) *rec*A1 *end*A1 *rel*A1 *deo*R96	Laboratory collection
*E. coli* BL21 (DE3)	F^–^ *omp*T *gal dcm lon hsd*SB (rB^−^ mB^−^) λ(DE3)	Laboratory collection
*E. coli* LGE2	*E. coli* BL21 (DE3) harboring pET-*lgk* and pZBC	Ref. [17]
*E. coli* + *lgk*	*E. coli* BL21 (DE3) harboring pET-*lgk*	Ref. [17]
*E. coli* Δ*xylF*	Chromosome gene *xylF*-deleted *E. coli* BL21 (DE3)	This study
*E. coli* Δ*kgtP*	Chromosome gene *kgtP*-deleted *E. coli* BL21 (DE3)	This study
*E. coli* Δ*xylF* + *lgk*	*E. coli* Δ*xylF* harboring pET-*lgk*	This study
*E. coli* Δ*kgtP* + *lgk*	*E. coli* Δ*kgtP* harboring pET-*lgk*	This study
*E. coli* Δ*xylF* + *lgk* + *xylF*	*E. coli* Δ*xylF* harboring pET-*xylF*-*lgk*	This study
*E. coli* Δ*kgtP* + *lgk* + *kgtP*	*E. coli* Δ*kgtP* harboring pET-*kgtP*-*lgk*	This study
Plasmids		
pET-21a	ColE1 ori, f1 ori, Amp^r^, T7*lac* promoter	Laboratory collection
pET-*lgk*	pET-21a vector carrying a codon-optimized *lgk* gene derived from *L. starkeyi*	Laboratory collection
pCasPA	oriV, oriT, araC, araBAD promoter, T7 promoter, lac operator, λRed genes (Gam, Beta, Exo), SacB, Cas9, Tet^R^, trfA	Purchased from Addgene repository
pACRISPR	pRO1600 oriV, ori, f1 ori, Amp^r^, T7 promoter, *trc* promoter, SP6 promoter, lac operator, gRNA scaffold, SacB	Purchased from Addgene repository
pACRISPR-sgRNAxylF	pACRISPR inserted with the sgRNA for gene *xylF* recognition	This study
pACRISPR-sgRNA-HRxylF	pACRISPR-sgRNAxylF inserted with the homologous arms for gene *xylF* deletion	This study
pACRISPR-sgRNAkgtP	pACRISPR inserted with the sgRNA for gene *kgtP* recognition	This study
pACRISPR-sgRNA-HRkgtP	pACRISPR-sgRNAxylF inserted with the homologous arms for gene *kgtP* deletion	This study
pET-*xylF-lgk*	pET-*lgk* vector carrying gene *xylF*	This study
pET-*kgtP-lgk*	pET-*lgk* vector carrying gene *kgtP*	This study
Primers		
F1 (for sgRNA of *xylF*)	5′-GTGGGTCACATCGATCGGTGTCAGG-3′	This study
R1 (for sgRNA of *xylF*)	5′-AAACCCTGACACCGATCGATGTGAC-3′	This study
F2 (for sgRNA of *kgtP*)	5′-GTGGGTTCCTGATGCGCCCAATAGG-3′	This study
R2 (for sgRNA of *kgtP*)	5′-AAACCCTATTGGGCGCATCAGGAAC-3′	This study
F3 (for left arm of *xylF*)	5′-ACGTCTAGAGACAGCGTAGCGTCATCAGG-3′	This study
R3 (for left arm of *xylF*)	5′-ATCCTCGAGGCTAGCGCCTCCTGACACCGAT-3′	This study
F4 (for right arm of *xylF*)	5′-TAGGCTAGCGCAGCAACGTTGGTAAGCAG-3′	This study
R4 (for right arm of *xylF*)	5′-CAGCTCGAGTGACGGAATGCTAACGGGT-3′	This study
F5 (for left arm of *kgtP*)	5′-ACGTCTAGAGAATTTGCCTGGCGG-3′	This study
R5 (for left arm of *kgtP*)	5′-ATCCTCGAGGCTAGCGCGACGTGTATCA-3′	This study
F6 (for right arm of *kgtP*)	5′-TAGGCTAGCTGATGGCCGTGGTG-3′	This study
R6 (for right arm of *kgtP*)	5′-CAGCTCGAGAGGTTCGTAAACTCATCCG-3′	This study
F7 (for restoration of *xylF*)	5′-CATGAATTCTATATCTCCTTCTTAAAGTTAATTAC AGCTCGCTCTC-3′	This study
R7 (for restoration of *xylF*)	5′-CGCGGATCCACCATGAAAATAAAG-3′	This study
F8 (for restoration of *kgtP*)	5′-CATGAATTCTATATCTCCTTCTTAAAGTTAACTAA AGACGCATC-3′	This study
R8 (for restoration of *kgtP*)	5′-CGCGGATCCATGGCTGAAAGT-3′	This study

Underlined regions of the primer sequences are restriction sites

### Samples preparation, proteins extraction, and peptides separation

The *E. coli* cells grown in M9 minimal medium supplied with either levoglucosan or fructose were harvested at both the early- and mid-logarithmic growth phase, with the respective OD_600_ value of 0.23 ± 0.02 and 0.57 ± 0.05 for proteomics analysis. The optical cell density was measured using a UV spectrophotometer (Unico Instrument Co., Ltd., Shanghai, China). All the experiments were conducted in triplicate. The harvested cells were washed and collected by centrifugation for protein extraction. The collected cells were lysed, reduced, alkylated, and digested as described previously [49]. The peptide mixture was fractionated by high pH reverse phase separation using LC-20AB HPLC system (Shimadzu, Japan) and then collected and dried in a vacuum concentrator (Christ RVC 2-25, Christ, Germany) for downstream analysis.

### Spectral library generation

Data-dependent acquisition (DDA) analysis was performed on a Q Exactive HF mass spectrometer (Thermo Fisher Scientific, San Jose, California) equipped with an EASY-nLC 1200 system (Thermo Fisher Scientific, San Jose, California). Data were acquired with full scans (*m*/*z* 300–1400) using an Orbitrap mass analyzer. The top 20 precursor ions were fragmented and transferred into the Orbitrap analyzer operating at a resolution of 15,000 at *m*/*z* 200. The automatic gain control (AGC) was set to 3e^6^ for full MS and 5e^4^ for MS/MS, with maximum ion injection times of 80 and 100 ms, respectively. Dynamic exclusion was set at 1/2 of peak width. DIA analysis was performed using the same system and parameters for DDA. The DIA scans were set at a resolution of 30,000, NCE of 27%, AGC target of 1e^6^, and maximal injection time of 45 ms. Fifty windows were set for DIA acquisition, ranging from 400 to 1200 *m*/*z*, using an isolation width of 16 *m*/*z*.

### Data analysis and bioinformatics analysis

Protein identification and quantification were conducted with the Spectronaut pulsar X 12.0 (Biognosys, Boston). First, the DDA raw files were searched in the Spectronaut pulsar against the *E. coli* BL21 (DE3) UniProt database (http://www.uniprot.org/uniprot/) to generate a spectral library using BGS factory settings. Peptides FDR was all set as 1%, and the iRT calibration *R*^2^ was 0.8. Next, the DIA data were analyzed for protein quantification. With the iRT regression typeset as local regression, all the results were filtered by a *Q* value cutoff of 0.01 (FDR of 0.01). The *p* value was estimated by Density Estimator and further adjusted by Bonferroni correction.

The paired difference test was used to identify DEPs. Proteins with log_2_FC > 1 or < − 1 (FC, fold change) and Bonferroni-adjusted *p* value < 0.05 were defined as DEPs. Functional enrichment of these DEPs was conducted by KEGG, GO, COG, and UniProt analysis.

### Real-time quantitative PCR

To study the mRNA levels in response to different carbon and energy sources, qPCR was performed. RNA was isolated using the TRIzol (Takara) according to the manufacturer’s protocol, followed by treatment with DNase I. qPCR was performed using the real-time fluorescence detection method on an Applied Biosystems 7300 system. The qPCR reaction volume was 20 μL, containing 10 μL 2× SYBR Green Real-Time PCR Master Mix, 0.4 μL forward primer (10 μM), 0.4 μL reverse primer (10 μM), 1 μL template cDNA (20 ng/μL), and 8.2 μL ddH_2_O. The primer pairs used are listed in Additional file 2: Table S2. The qPCR condition was set as 2 min at 95 °C; 40 cycles of 15 s at 95 °C, 30 s at the respective annealing temperature (Additional file 2: Table S2), 25 s at 72 °C; followed by a melting curve for 15 s at 95 °C, 60 s at 60 °C, and finally for 15 s at 95 °C. Each sample was performed in triplicate. The 16 s rRNA gene was used as the endogenous housekeeping gene [50]. Data were analyzed using the 2^−ΔΔCT^ method to evaluate the transcriptional fold change level, with the Ct threshold set automatically by the system for all samples.

### Plasmid construction for the genome editing of *E. coli* BL21 (DE3)

A genome editing system coupled with the λ Red recombination system [51] was used to improve the mutation efficiency. A suitable 10-bp spacer sequence annealed by sgRNA primers listed in (Table 4) before the PAM site (NGG) of the target locus (*xylF* or *kgtP*) was chosen as the guide sequence for gene deletion using the online design tool (http://crispor.org). Then, the corresponding sgRNAs were synthesized by Sangon Biotech (Shanghai) Co., Ltd, and subsequently phosphorylated, annealed, and inserted into the BsaI sites of the pACRISPR plasmid to generate the pACRISPR-sgRNA plasmids. About 500-bp sequence (designed as left homologous arm) upstream of 5’ end of the gene *xylF* or *kgtP* was amplified using restriction sites-containing primer pairs F3/R3 or F5/R5 (Table 4). The resulting left homologous arm was flanked by XbaI and NdeI–XhoI sites, in which the NdeI site was intentionally introduced in the system because restriction sites in the original pACRISPR plasmid are limited. Then, about 500-bp sequence (designed as right homologous arm) downstream of 3′ end of the gene *xylF* or *kgtP* was amplified using primer pairs F4/R4 or F6/R6. The resulting right homologous arm was flanked by NdeI and XhoI sites. The left and right homologous arms were sequentially inserted into the corresponding pACRISPR-sgRNA plasmids to generate pACRISPR-sgRNA-HRxylF and pACRISPR-sgRNA-HRkgtP plasmids, respectively, which were used for the efficient deletion of genes *xylF* and *kgtP* of *E. coli* BL21 (DE3). Competent cells of *E. coli* were prepared by the CaCl_2_ method. PCR amplification, plasmid DNA extraction, DNA ligation was executed according to previously described procedures [17].

### Gene knockout using the pCasPA/pACRISPR system

At least 1 μg of pCasPA plasmid was transformed into 100 μL *E. coli* BL21 (DE3) competent cells using electroporation with the parameters of 1100 V, 400 Ω, 6 μF, and 2 mm cuvette of a Scientz-2C gene pulser. The pCasPA-containing colony was selected on LB agar plates added with 15 μg/mL tetracycline, confirmed by PCR, and cultured in the LB medium at 37 °C and 150 rpm. Once the OD_600_ of the culture reached about 0.2, a final concentration of 30 mM l-arabinose was added to the culture to induce the expression of the Cas9 nuclease and the λ-Red system. After another 2-h incubation, the culture was harvested to prepare the competent cells. Next, the pACRISPR-sgRNA-HRxylF and pACRISPR-sgRNA-HRkgtP plasmids assembled with the spacer and repair template were electroporated into the competent cells. Transformed cells were recovered in LB media at 37 °C for 1 h and plated onto the LB agar plate containing 15 μg/mL tetracycline and 100 μg/mL ampicillin. PCR and sequencing were used to verify the mutants and evaluate the genome editing efficiency. The successful knockout strains of *xylF* and *kgtP* were named *E. coli* Δ*xylF* and *E. coli* Δ*kgtP*, respectively.

### Gene restoration of *xylF* and *kgtP* in the mutated *E. coli*

The plasmid pET-*lgk* previously constructed in our laboratory [17], was used as the donor of gene *lgk*, and to restore genes *xylF* and *kgtP*. Gene *xylF* was amplified from *E. coli* BL21 (DE3) genomic DNA using primers F7 and R7 containing BamHI/EcoRI restriction sites (Table 4). Then, the sequenced *xylF* fragment was digested with BamHI/EcoRI and cloned into the pET-*lgk* plasmid to generate a pET-*xylF-lgk* plasmid. In this process, the ribosome binding site sequence corresponding to the T7*lac* promoter was added to the 3′ downstream of *xylF*. Gene *kgtP* was also amplified from *E. coli* BL21 (DE3) using primers F8 and R8 (Table 4). The construction of the pET-*kgtP-lgk* plasmid followed the same procedure as that of the pET-*xylF-lgk* plasmid. Finally, the pET-*xylF-lgk* and pET-*kgtP-lgk* plasmids were introduced into the competent cells of *E. coli* Δ*xylF* and Δ*kgtP*, respectively, to generate the gene-restored strains *E. coli* Δ*xylF* + *lgk* + *xylF* and *E. coli* Δ*kgtP* + *lgk* + *kgtP*. In parallel, pET-*lgk* plasmid was introduced into the competent cells of *E. coli* Δ*xylF* and *E. coli* Δ*kgtP*, respectively, to generate control strains *E. coli* Δ*xylF* + *lgk* and *E. coli* Δ*kgtP* + *lgk* to test the effect of *xylF* or *kgtP* deletion on sugar substrate utilization, especially the levoglucosan utilization.

### Cell growth and sugars utilization tests

*Escherichia coli* BL21 (DE3), *E. coli* (pET-*lgk*), *E. coli* Δ*xylF*, *E. coli* Δ*kgtP*, *E. coli* Δ*xylF* + *lgk*, *E. coli* Δ*kgtP* + *lgk*, *E. coli* Δ*xylF* + *lgk* + *xylF*, *E. coli* Δ*kgtP* + *lgk* + *kgtP* strains were individually inoculated into 100-mL M9 minimum media supplemented with 1% (w/v) levoglucosan and fructose, respectively. For each time interval, 5-mL culture media were taken and centrifuged to separate the cells and supernatants. *E. coli* cells were pelleted by centrifugation at 6000 rpm for 5 min, washed twice, and then re-suspended in 5-mL ice-cold water. Cell density was detected by a UV-2000 spectrophotometer set at *λ* = 600 nm. After centrifugation, the harvested cell pellets were placed in an oven set at 70 °C to determine the dry cell weights; the clarified supernatants were used for sugar analysis.

### Molecular docking of ABC transporter XylF and levoglucosan

Lamarckian genetic algorithm of AutoDock 4.2 software was used for molecular docking. The structure file of target protein XylF was obtained from PDB database (PDB_ID: 3MA0), and the structure file of target sugar levoglucosan was drawn by Chem3D software. The AutoDock software was used to add H atoms, add Gasteiger-Hücker empirical charges, combine non-polar hydrogen and set rotatable bonds. σ bonds between heavy atoms in the structure of levoglucosan were all set as rotatable bonds, and XylF was regarded as rigid structures. During the docking process, a 60 × 60 × 60-step docking square box (step length 0.375 Å) was set at the binding site of XylF, and levoglucosan was independently docked at the binding site for 200 times. Lamarckian genetic algorithm generated 150 random orientation and random small molecule conformations, and each round of passage was optimized for up to 1,500,000 times of energy optimization. The optimal ten conformations were selected for passage, the gene exchange rate of passage was 0.8 and the mutation rate was 0.02. The calculation was terminated after 27,000 generations of optimization. Other parameters used to run the program were set to default values in AutoDock 4.2 software. After docking, cluster analysis of the 200 docking results was performed, and the binding conformation with the best docking score (the lowest scoring value) was selected from the optimal cluster, to determine the binding site and binding mode of XylF and levoglucosan.

### Analytical method

Analyses of fructose and levoglucosan were performed using a high-performance liquid chromatography system (HPLC, LC-20AT, Shimadzu Corporation) described previously [2]. The specific growth rate *μ* was calculated using the dry cell weights detected at different time points [17]. Three replicate samples were evaluated in each case. All reagents used in this study were of analytical grade.

## Supplementary Information

**Additional file 1.** A total of 2749 proteins identified in the study.

**Additional file 2: Text S1.** Detailed analysis of the distribution and enrichment of the DEPs based on GO and KEGG analysis, respectively. **Text S2.** Proteins involved in carbohydrate metabolism and energy production were significantly differentially expressed. **Table S1.** Parameters of hydrogen bond length and angle between levoglucosan and XylF. **Table S2.** Primers used in the qPCR experiments. **Figure S1.** The distribution and enrichment of DEPs based on Gene Ontology (GO). **Figure S2.** Enrichment of KEGG pathway for the DEPs at early-log phase (A) and mid-log phase (B). **Figure S3.** The protein–protein interactions and involved biological processes of the DEPs related to carbonhydrate metabolism and energy production and conversion. **Figure S4.** Plates screening and PCR verification of the gene knockout strains. **Figure S5.** Map of plasmid pCasPA. **Figure S6.** Map of plasmid pACRISPR.

## Data Availability

The datasets used and/or analyzed during the current study are available from the corresponding author on reasonable request.

## Abbreviations

ABC: ATP-binding cassette
MFS: Major facility superfamily
LGK: Levoglucosan kinase
CCR: Carbon catabolite repression
DEPs: Differentially expressed proteins
DDA: Data-dependent acquisition
DIA: Data-independent acquisition
KEGG: Kyoto Encyclopedia of Genes and Genomes
GO: Gene Ontology
COG: Cluster of Orthologous Groups
A: Cells treated by acetic acid
F: Cells treated by furfural
P: Cells treated by phenol
C: Cells treated by combined inhibitors
MIC: Minimum inhibitory concentration
OD_600_: Optical density at wavelength (*λ*) 600 nm
HPLC: High-performance liquid chromatography
G6P: Glucose-6-phosphate
PEP: Phosphoenolpyruvate
PTS: Phosphotransferase system
F1P: Fructose 1-phosphate
F6P: Fructose 6-phosphate
CRP: Cyclic AMP receptor protein
