# Supplementary material for: Omics analysis coupled with gene editing revealed potential transporters and regulators related to levoglucosan metabolism efficiency of the engineered Escherichia coli
Source: Biotechnol Biofuels Bioprod. 2022 Jan 11;15:2. doi: 10.1186/s13068-022-02102-4 (PMC8753852; doi:10.1186/s13068-022-02102-4)
Supplement: Supplementary file 2 — Additional file 2: Text S1. Detailed analysis of the distribution and enrichment of the DEPs based on GO and KEGG analysis, respectively. Text S2. Proteins involved in carbohydrate metabolism and energy production were significantly differentially expressed. Table S1. Parameters of hydrogen bond length and angle between levoglucosan and XylF. Table S2. Primers used in the qPCR experiments. Figure S1. The distribution and enrichment of DEPs based on Gene Ontology (GO). Figure S2. Enrichment of KEGG pathway for the DEPs at early-log phase (A) and mid-log phase (B). Figure S3. The protein–protein interactions and involved biological processes of the DEPs related to carbonhydrate metabolism and energy production and conversion. Figure S4. Plates screening and PCR verification of the gene knockout strains. Figure S5. Map of plasmid pCasPA. Figure S6. Map of plasmid pACRISPR. [file 13068_2022_2102_MOESM2_ESM.docx]

**Omics analysis coupled with gene editing revealed potential transporters and regulators related to levoglucosan metabolism efficiency of the engineered *Escherichia* coli**

Dongdong Chang^1^, Cong Wang^1^, Zia Ul Islam^1, 2^, Zhisheng Yu^1,3^*

1. College of Resources and Environment, University of Chinese Academy of Sciences, Beijing 100049, P. R. China

2. Department of Chemistry, Mississippi State University, Mississippi State, MS 39762, USA.

3. RCEES-IMCAS-UCAS Joint-Lab of Microbial Technology for Environmental Science, Beijing 100085, P.R. China

All correspondence should be addressed to:

Prof. Zhisheng Yu

College of Resources and Environment

University of Chinese Academy of Sciences,

Beijing 100049, P. R. China

E-mail: [yuzs@ucas.ac.cn](mailto:yuzs@ucas.ac.cn)

Tel.: +86 10 88256057

**This additional file contains: Text S1** Detailed analysis of the distribution and enrichment of the DEPs based on GO and KEGG analysis, respectively**; Text S2** Proteins involved in carbohydrate metabolism and energy production were significantly differentially expressed; **Table S1** Parameters of hydrogen bond length and angle between levoglucosan and XylF; **Table S2** Primers used in the qPCR experiments; **Fig. S1** The distribution and enrichment of DEPs based on Gene Ontology (GO); **Fig. S2** Enrichment of KEGG pathway for the DEPs at early-log phase (A) and mid-log phase (B); **Fig. S3** The protein-protein interactions and involved biological processes of the DEPs related to carbonhydrate metabolism and energy production and conversion; **Fig. S4** Plates screening and PCR verification of the gene knockout strains; **Fig. S5** Map of plasmid pCasPA; **Fig. S6** Map of plasmid pACRISPR.

**Text S1 Detailed analysis of the distribution and enrichment of the DEPs based on GO and KEGG analysis, respectively**

Based on the GO analysis tool, the DEPs were classified into three categories: biological processes (BP), cellular component (CC), and molecular function (MF). The upregulated and downregulated protein numbers of the DEPs distributed in the main sub-categories of the GO categories are shown in Additional file 2, Fig. S1A for the early-log phase. In parallel, those for the mid-log phase are shown in Additional file 2, Fig. S1B. The results show that the DEPs identified at both early- and mid-log phases are mainly involved in the metabolic process (GO:0008152), cellular process (GO:0009987), and localization process (GO:0051179) for BP category; in cell (GO:0005623), cell part (GO:0044464), and membrane (GO:0016020) for CC category; in catalytic activity (GO:0003824), binding (GO:0005488), and transporter activity (GO:0005215) for MF category. Further enrichment-based clustering of GO terms for the DEPs at early- and mid-log phases are exhibited in Additional file 2, Figs. S1C and S1D, respectively, which show that the DEPs were significantly (Bonferroni-adjusted *p*-value＜0.05) enriched in localization (GO: 0051179), transport (GO:0006810), and establishment of localization process (GO:0051234) for BP category; membrane (GO:0016020), an integral component of membrane (GO:0016021), and an intrinsic component of membrane (GO:0031224) for CC category; transmembrane transporter activity (GO:0022857) and transporter activity (GO:0005215) for MF category.

The KEGG pathway analysis showed that metabolic pathways (ebe01100), ABC transporters (ebe02010), carbon metabolism (ebe01200), microbial metabolism in diverse environments (ebe01120), biosynthesis of secondary metabolites (ebe01110), biosynthesis of antibiotics (ebe01130), fructose and mannose metabolism (ebe00051), two-component system (ebe02020), and biosynthesis of amino acids (ebe01230), are the main DEPs involving pathway. Enrichments of KEGG pathway for the DEPs show that the significantly enriched pathways (Bonferroni-adjusted *p*-value＜0.05) for early-log phase are fructose and mannose metabolism (ebe00051), two-component system (ebe02020), glycolysis/ gluconeogenesis pathways (ebe00010), bacterial chemotaxis (ebe02030), and microbial metabolism in diverse environments (ebe01120) (Additional file 2, Fig. S2A); while for mid-log phase were flagellar assembly (ebe02040), ABC transporters (ebe02010), fructose and mannose metabolism (ebe00051), fatty acid degradation (ebe01212), and phosphotransferase system (PTS) pathways (ebe02060) (Additional file 2, Fig. S2B).

**Text S2** **Proteins involved in carbohydrate metabolism and energy production were significantly differentially expressed**

Energy is fundamental to all lives. For the microbial utilization of a specific sugar, energy is consumed and produced through the whole metabolism process. PEP-PTS is the most efficient sugar-utilization system because only one mol of ATP (equivalent to one mol of PEP) is consumed for each mol of imported and phosphorylated sugar (Fig. 2. However, sugar transport by the ABC and MFS transporter is usually ATP-dependent, requiring two mol ATP or one mol of H^+^ and one mol of ATP for the transport and phosphorylation of one mole of sugar (Fig. 2).

As levoglucosan utilization is involved in the non-PTS way, more ATP would be required during the levoglucosan transport. Our results indicate that large numbers of proteins involved in carbohydrate metabolism and energy production and conversion were differentially expressed in response to levoglucosan metabolism to adjust the energy supply (Tables 1 and 2). For instance, DEPs related to carbohydrate metabolism like LGK (heterogeneous protein), XylA (A0A140N6S9), PpsA (A0A140NB77), TreC (A0A140NGD0), IdnK (A0A140NGU6), GntK (A0A140N6M3), and Gcl (A0A140NEP9) were upregulated at both phases; while AlsE (A0A140SS41), FbaA (A0A140N821), Epd (A0A140N827), and FruK (A0A140N679) were downregulated. In parallel, DEPs related to energy production and conversion like AstD (A0A140N931), YqhD (A0A140N5B5), GlcF (A0A140N897), and YdeM (A0A140N975) were upregulated, while YahF (A0A140NAI7), AdhE (A0A140NCE4), YgeT (A0A140N5N6), CyoC (A0A140NC92), FdhF (A0A140NE68), HyaA (A0A140NDP4), HyaB (A0A140NB83), ViaA (A0A140NFS7), and RavA (A0A140NI88) were downregulated.

Levoglucosan kinase LGK responsible for the phosphorylation of levoglucosan to glucose-6-phosphate was upregulated by 7.3 and 10.5 folds, respectively, due to cellular energy, compound synthesis, and other demands for survival in a solely levoglucosan-based media. Another vital enzyme, PEP synthase PpsA, involved in the central carbon metabolic pathway responsible for the generation of PEP, which is required to synthesize precursor metabolites for cellular carbon compounds, was upregulated by 4.8 and 3.7 folds at early- and mid-log phases, respectively. Moreover, the sugar metabolism-related enzymes like xylose isomerase XylA responsible for the catalytic conversion of D-xylose to D-xylulose and D-glucose to D-fructose, and trehalose-6-phosphate hydrolase TreC that can hydrolyze trehalose-6-phosphate into glucose and glucose-6-phosphate and convert maltose to maltose-1-phosphate, were upregulated by about 6.2 and 4.2 folds at early-log phase and 6.5 and 11.3 folds at mid-log phase, respectively. The gluconate metabolic pathway involved enzymes D-gluconate kinases IdnK and GntK responsible for the degradation of D-gluconate, L-idonate, and 5-ketogluconate were up-regulated by about 2.4 and 2.2 folds at early-log phase and 2.4 and 2.8 folds at mid-log phase, respectively. The glycolate and glyoxylate metabolic pathway involved enzyme glyoxylate carboligase Gcl was upregulated significantly with the values of respective about 14.2 and 12.2 folds, and in the meanwhile, the glycolate dehydrogenase GlcF that could enhance energy supply by supplementing metabolic intermediates for glyoxylate and TCA cycles through catalyzing glycolate to glyoxylate as well as an oxidized-state electron carrier to reduced state [33] was also upregulated by about 3.8 and 2.7 folds, respectively. Apart from GlcF, several energy supply-related oxidoreductases were also upregulated. For example, the succinyl glutamate semialdehyde dehydrogenase AstD that is activated with NAD^+^ as the electron acceptor and of broad substrate specificity [1], the NADPH-dependent aldehyde reductase YqhD as the major glycolaldehyde reductase that can facilitate the interconversion of NADP and NADPH [2], and the anaerobic sulfatase maturase YdeM as a member of the radical S-adenosyl-l-methionine enzyme superfamily enzymes with iron-sulfur cluster binding and oxidoreductase activity, were upregulated by about 6.2, 8.5, and 4.4 folds at early-log phase and 20.4, 6.5, and 2.1 folds at mid-log phase, respectively.

However, although the crucial enzyme PpsA involved in the glycolytic pathway was upregulated during levoglucosan metabolism when compared to fructose metabolism, allulose-6-phosphate 3-epimerase AlsE catalyzing the equilibration of the D-allulose 6-phosphate and D-fructose 6-phosphate, fructose-bisphosphate aldolase class II FbaA cleaving FBP to produce G3P and glycerone phosphate [3], D-erythrose-4-phosphate dehydrogenase Epd catalyzing the oxidation of erythrose 4-phosphate (E4P) and G3P, and phosphofructokinase FruK highly specific for F1P and essential for the utilization of fructose [4], were downregulated by about 2.3, 2.9, 3.9, and 12.7 folds at early-log phase and 16.8, 2.4, 2.5 and 14.0 folds mid-log phase, respectively. The relative downregulation of these carbohydrate metabolism-related proteins in levoglucosan-utilizing cells is within our expectations, as these proteins are primarily involved in the fructose metabolic pathway and undoubtedly expressed at a higher level in fructose-utilizing cells than in the levoglucosan-utilizing cells.

In addition, the membrane-bound respiratory modules are crucial for the cell flexibility in the H^+^/e^-^ ratio (the number of protons delivered to the periplasm per electron) and the rapid cell responsiveness in the ATP synthesis rate [5]. Cytochrome *bo*_3_ ubiquinol oxidase subunit 3 CyoC, involved in the respiratory chain of *E. coli* for proton pumping and electron transfer, was downregulated by 4.1 and 2.4 folds, respectively; this would increase the reduction of ubiquinone pool and thereby affect the activity of NADH dehydrogenase [5]. ViaA and RavA have interactions with CyoC (Additional file 2, Fig. S3). RavA and ViaA that can stimulate the ATPase activity of RavA together functioning as a regulatory chaperone for Fe-S cluster protein homeostasis and bacterial respiration [6] were downregulated by about 2.4 and 2.7 folds at early-mid phase and about 2.2 and 2.3 folds at log-mid phase, respectively. Also, as an acyl-CoA synthetase coupling the hydrolysis of succinyl-CoA to the synthesis of ATP or GTP and representing the only step of substrate-level phosphorylation in the TCA cycles [7], YahF was found downregulated by about 5.4 and 4.5 folds, respectively. Besides, four dehydrogenases like AdhE, FdhF, HyaA, and HyaB that has been documented to be interacted with PpsA (Additional file 2, Fig. S3) were also downregulated to adjust the NADH/NAD^+^ and H^+^/e^-^ ratios, which could play key roles in the ATP production and conversion and the sugar transport by sugar: proton H^+^ symporter. The aldehyde/alcohol dehydrogenase AdhE expression correlated with the NADH to NAD^+^ ratio in the cell [8] was downregulated by about 3.5 and 2.9 folds. Formate dehydrogenase-H FdhF as a membrane-associated formate dehydrogenase isoenzyme playing a role in the formate-dependent catabolism of urate [9], was downregulated by 2.3 and 7.1 folds, respectively. In *Escherichia coli*, hydrogen oxidation/uptake as an important feature of energy conservation is catalyzed by membrane-bound [NiFe] hydrogenase-1 and [NiFe] hydrogenase-2. HyaA and HyaB, as the respective small and large subunit of hydrogenase-1 involved in the hydrogen to trimethylamine N-oxide and dimethyl sulfoxide electron transfer pathways [10], were downregulated by about 4.3 and 2.1 folds at early-mid phase, and about 2.1 and 2.3 folds at log-mid phase, respectively. Another putative dehydrogenase YgeT that could keep the H^+^ balance and catalyze the interconversion of xanthine to urate and NADH to NAD^+^ [11], was also downregulated by 8.1 and 2.1 folds, respectively. All the results suggested that energy supply during levoglucosan metabolism was globally regulated by different biological pathways.

**References**

1. Butland G, Peregrín-Alvarez JM, Li J, Yang W, Yang X, Starostine A, Richards D, Beattie B, Krogan N, Davey M. Interaction network containing conserved and essential protein complexes in *Escherichia coli.* Nature, 2005; 433:531-537.

2. Alkim C, Cam Y, Trichez D, Auriol C, Spina L, Vax A, Bartolo F, Besse P, François JM, Walther T. Optimization of ethylene glycol production from (D)-xylose via a synthetic pathway implemented in *Escherichia coli.* Microb Cell Fact, 2015; 14:127.

3. Katebi AR and Jernigan RL. Aldolases utilize different oligomeric states to preserve their functional dynamics*.* Biochemistry, 2015; 54:3543-3554.

4. Aboulwafa M, Zhang Z, Saier Jr MH. Protein-Protein Interactions in the Cytoplasmic Membrane of *Escherichia coli*: Influence of the Overexpression of Diverse Transporter-Encoding Genes on the Activities of PTS Sugar Uptake Systems*.* Microb Physiol, 2020; 30:36-49.

5. Sharma P, Hellingwerf KJ, de Mattos MJ, Bekker M. Uncoupling of substrate-level phosphorylation in *Escherichia coli* during glucose-limited growth*.* Appl Environ Microbiol, 2012; 78:6908-13.

6. Wong KS, Snider JD, Graham C, Greenblatt JF, Emili A, Babu M, Houry WA. The MoxR ATPase RavA and its cofactor ViaA interact with the NADH:ubiquinone oxidoreductase I in *Escherichia coli.* PLoS One, 2014; 9:e85529.

7. Sandoval NR, Kim JYH, Glebes TY, Reeder PJ, Aucoin HR, Warner JR, Gill RT. Strategy for directing combinatorial genome engineering in Escherichia coli*.* PNAS, 2012; 109:10540-10545.

8. Peng L and Shimizu K. Global metabolic regulation analysis for *Escherichia coli* K12 based on protein expression by 2-dimensional electrophoresis and enzyme activity measurement*.* Applied Microbiology Biotechnology, 2003; 61:163-178.

9. Iwadate Y and Kato JI. Identification of a Formate-Dependent Uric Acid Degradation Pathway in *Escherichia coli.* J Bacteriol, 2019.

10. Dubini A, Pye RL, Jack RL, Palmer T, Sargent F. How bacteria get energy from hydrogen: a genetic analysis of periplasmic hydrogen oxidation in *Escherichia coli.* Int J Hydrogen Energy, 2002; 27:1413-1420.

11. Xi H, Schneider BL, Reitzer L. Purine Catabolism in *Escherichia coli* and Function of Xanthine Dehydrogenase in Purine Salvage*.* J Bacteriol, 2000; 182:5332-5341.

**Table S1** **Parameters of hydrogen bond length and angle between levoglucosan and XylF**

| Index | Residue | Distance H-A (Å) | Distance D-A (Å) | Donor Angle (°) |
| --- | --- | --- | --- | --- |
| 1 | ASP-90 | 1.62 | 2.58 | 165.22 |
| 2 | ARG-91 | 2.22 | 3.03 | 136.5 |
| 3 | ARG-91 | 3.06 | 4.01 | 156.87 |
| 4 | ASP-135 | 1.63 | 2.61 | 171.72 |
| 5 | ASN-137 | 2.48 | 3.38 | 147.26 |
| 6 | ASN-196 | 2.28 | 3.06 | 133.57 |
| 7 | LYS-242 | 2.13 | 2.89 | 130.09 |

Note: Distance H-A denotes the distance between hydrogen atom (H) and acceptor atom (A); Distance D-A denotes the distance between donor atom (D) and acceptor atom (A)

**Table S2** **Primers used in the qPCR experiments**

| Gene Name | Forward Primer | Tm | GC% | Reverse Primer | Tm | GC% | Product Length | Product TaOpt |
| --- | --- | --- | --- | --- | --- | --- | --- | --- |
| *xylF* | CCGTTGAATTGGGCAATGGTCA | 58.1 | 50 | GCTCGCTCTCTTTGTGGAATCC | 57.7 | 54.5 | 156 | 54.8 |
| *malE* | CGGTCTCGCTGAAGTCGGTAA | 58.1 | 57.1 | CGGTCGTGTGCCCAGAAGATA | 58.1 | 57.1 | 144 | 55.7 |
| *ugpB* | CAGCTACGTCGGTCGTAAGGAT | 58.1 | 54.5 | ATCGGCGTCGTAAGGCATCAT | 58.2 | 52.4 | 148 | 56.4 |
| *ugpC* | GCGATGGTGTTCCAGAACTACG | 57.8 | 54.5 | ACCGTCCAGCTCCAGAATGC | 58.1 | 60 | 144 | 57 |
| *mglA* | GGTTCGCTCTCCGGTGGTAAT | 58 | 57.1 | CCTTGCCTTTCTTCGCCAGTTC | 58.4 | 54.5 | 160 | 55.1 |
| *kgtP* | TGATGTGTGCCCTGCTGATAGT | 57.8 | 50 | GTACTCCGCCGAACCACCAA | 58.1 | 60 | 146 | 55.1 |
| *lamB* | GTCTTCGCAACCTACGCCAAG | 57.8 | 57.1 | AAGGTCCACTCGTCGCTGTC | 57.8 | 60 | 146 | 57.3 |
| *gntT* | TGCTGATTGCGATGTTCACCTT | 57.2 | 45.5 | TTGTCCACGCCGCTGTCTAC | 58.5 | 60 | 160 | 55.6 |
| *xylA* | CGACGAACCCAGATCCTGAAGT | 58.1 | 54.5 | TTCACGCTCCTGACGCAAGT | 58 | 55 | 158 | 56.5 |
| *galS* | TCGCCCAGCAGCATCAGAAA | 58.1 | 55 | CGCCAGTTCATCGTCACTCAAT | 57.4 | 50 | 146 | 54.6 |
| *malM* | AACTGACCCTGACGCTGACC | 57.7 | 60 | CATCACGCCTGGTTCCTGGTA | 58 | 57.1 | 140 | 55.7 |
| *fruA* | TGGTGATTGTGGCGGCAGATA | 57.9 | 52.4 | GGTTCATACGGCGTTGCTTCA | 57.5 | 52.4 | 142 | 55.9 |
| *fruB* | GCCATCAATGAACAACCGCTGA | 58.2 | 50 | CGTCATTCATCGCCACACTCAC | 58.3 | 54.5 | 160 | 57.9 |
| *fruK* | GGCTCGGTCAGTTCGATATGGT | 58.3 | 54.5 | GCTACTAACGCTTCACGGCTAC | 57.6 | 54.5 | 142 | 56.7 |
| *hyaA* | TACTCATTCCACCGCCGATACC | 58 | 54.5 | CCTCATTGCCTGGCTGATGTTC | 58 | 54.5 | 149 | 58.4 |
| *viaA* | GACGGTTCCTGAGCAGGTTGA | 58.2 | 57.1 | AGGCGATAGGTGAGCAACTGTT | 58.1 | 50 | 147 | 55.9 |
| *yahF* | CCGCAGCATCAAACACACCTT | 57.9 | 52.4 | ATCACGATCACCGCCACTTCT | 57.7 | 52.4 | 141 | 57.1 |


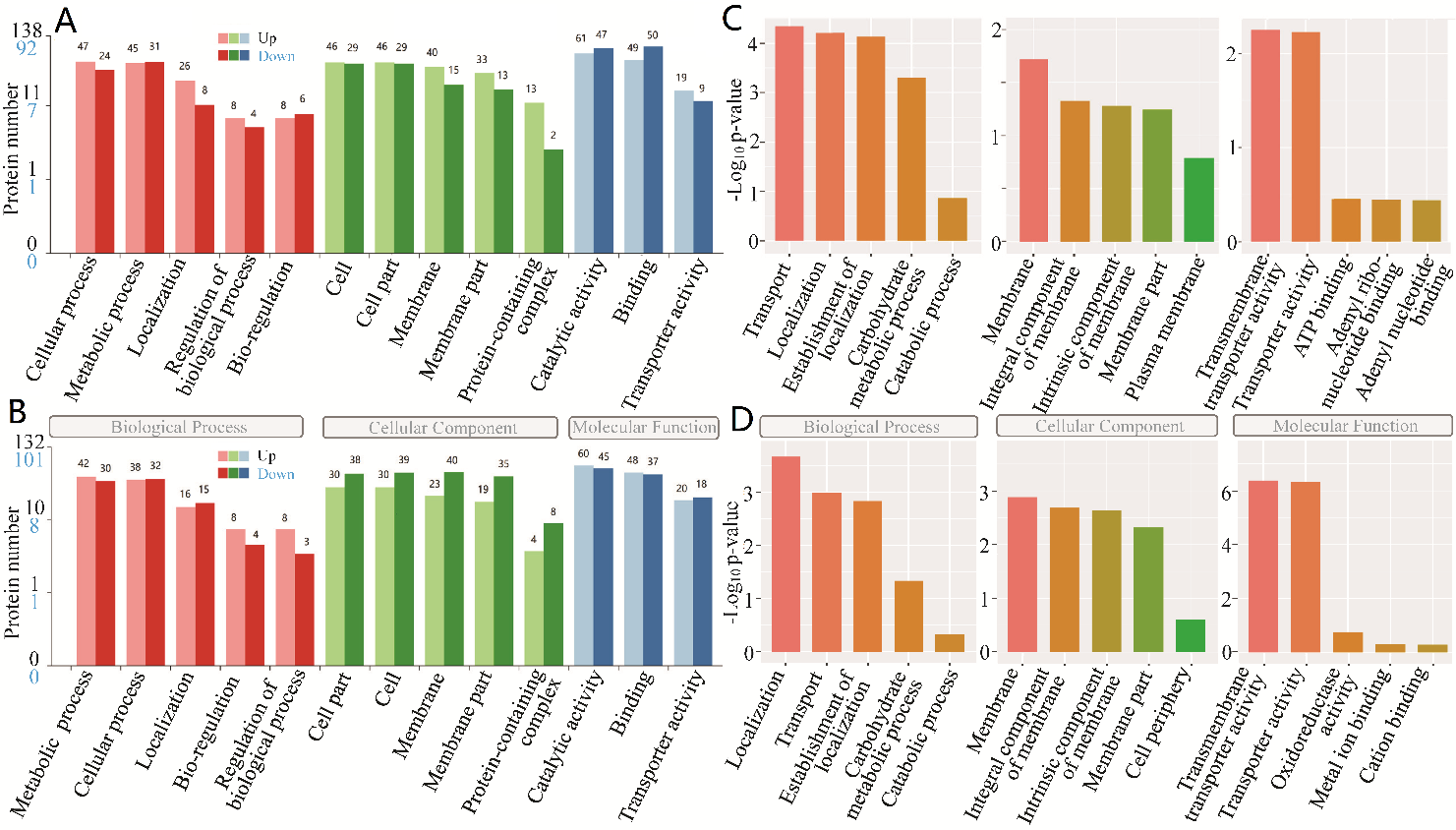


**Fig. S1 The distribution and enrichment of DEPs based on Gene Ontology (GO).** *E. coli* LGE2 cells were cultured at 37°C and 150 rpm in levoglucosan- and fructose-based M9 minimal media, and then harvested at both the early- and mid-log phases. The upregulated and downregulated protein numbers of the DEPs distributed in the main sub-categories of the GO categories were listed in **A)** for the early-log phase and **B)** for the mid-log phase. The enrichment clustering of GO terms for the DEPs were listed in **C)** for the early-log phase and **D)** for the mid-log phase.


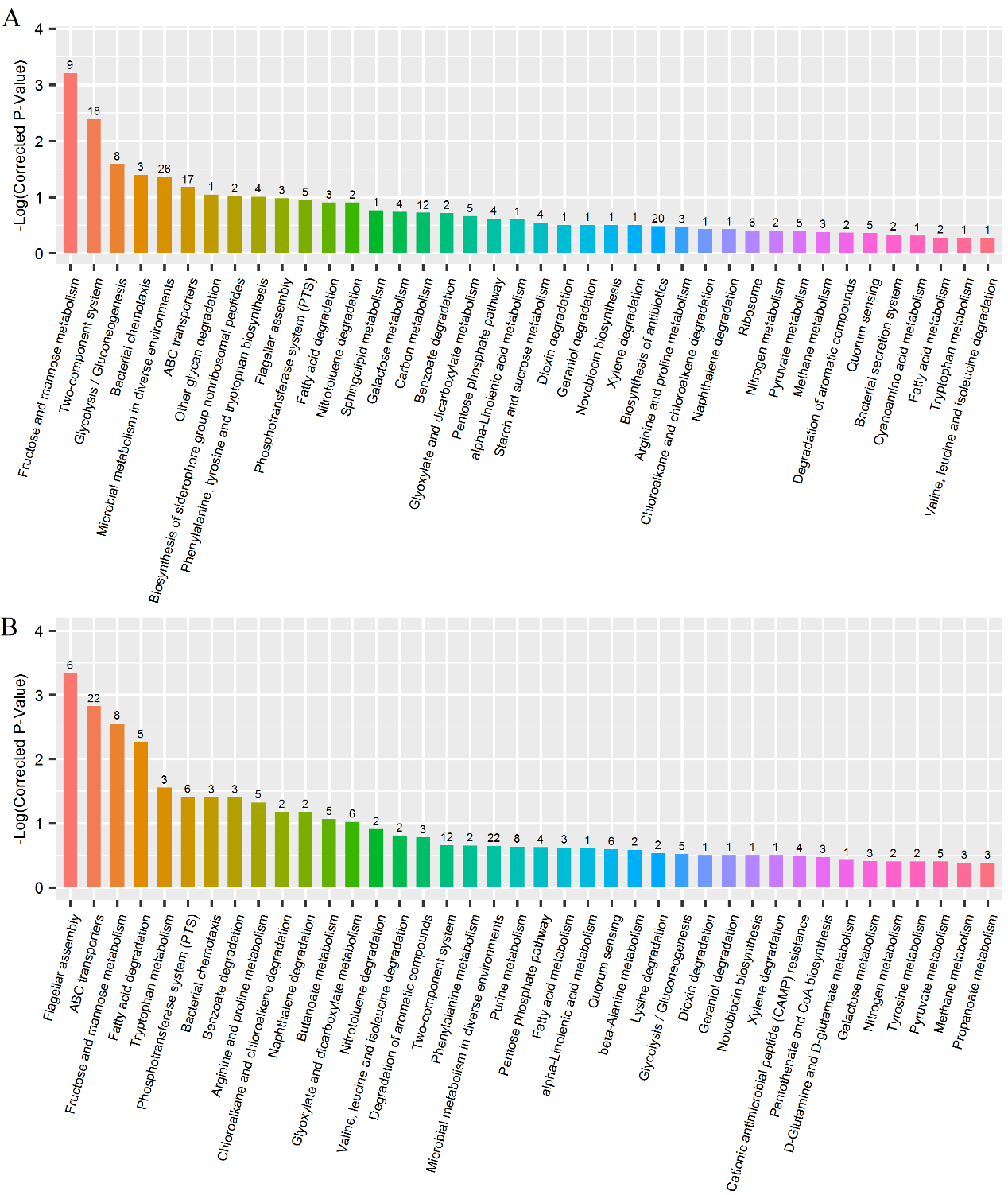


**Fig. S2 Enrichment of KEGG pathway for the DEPs at early-log phase (A) and mid-log phase (B)**


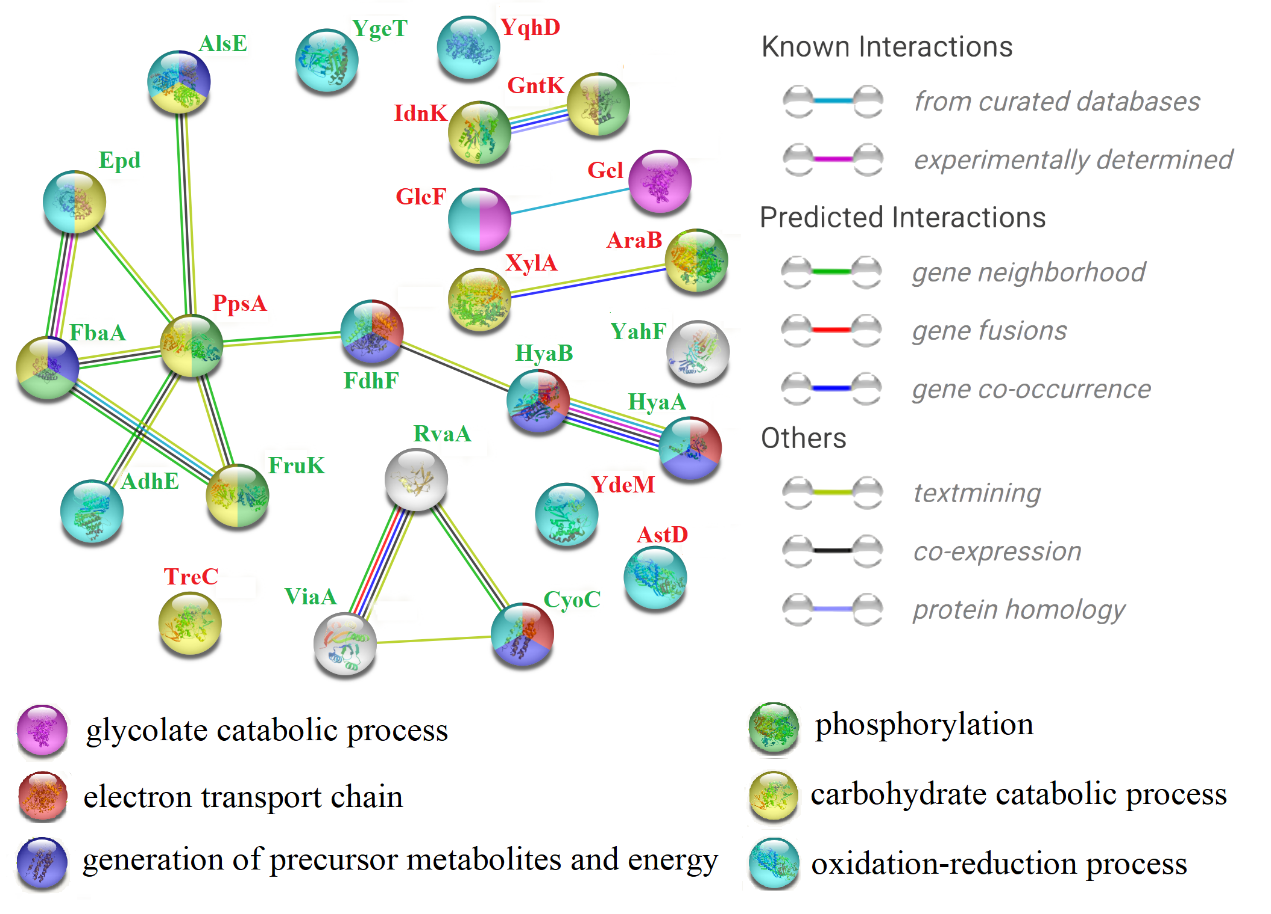


**Fig. S3 The protein-protein interactions and involved biological processes of the DEPs related to carbonhydrate metabolism and energy production and conversion**


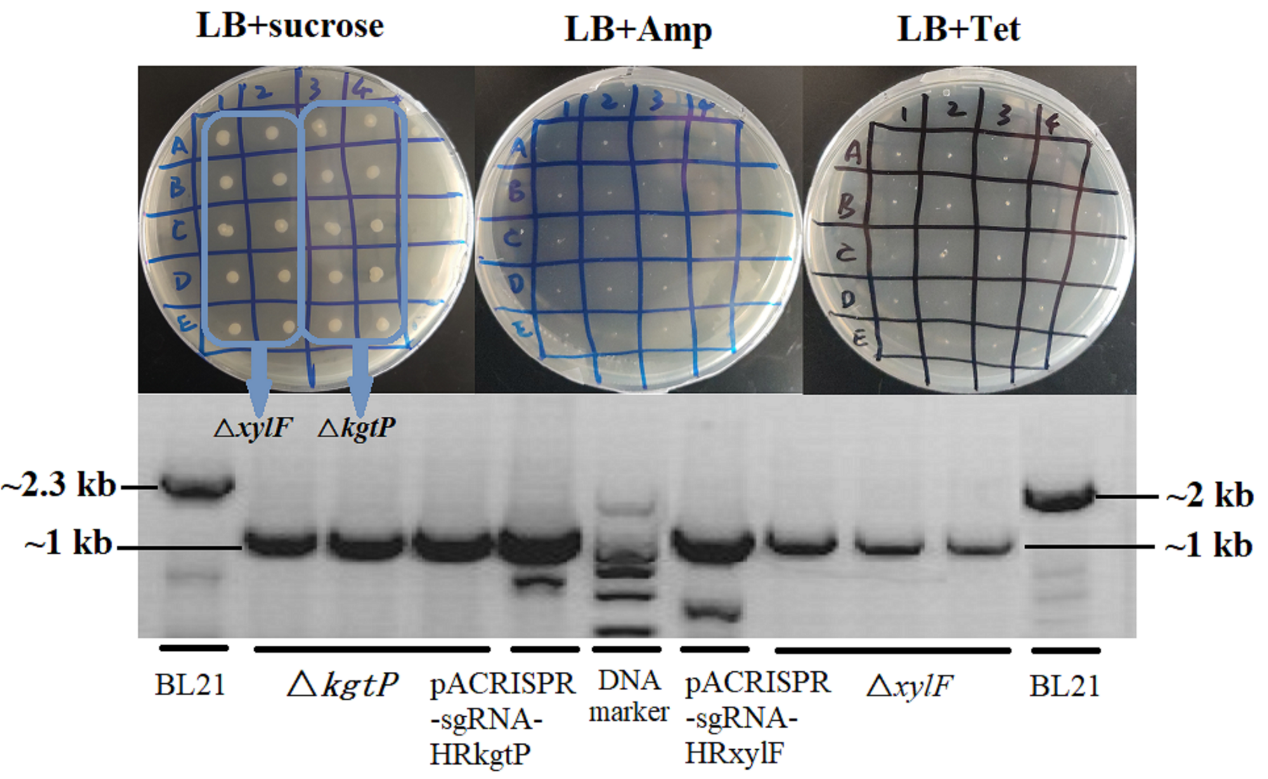


**Fig. S4. Plates screening and PCR verification of the gene knockout strains.** The gene-edited strains were firstly spread on sucrose-containing LB plates to eliminate the pCasPA and pACRISPR-derived plasmids by sucrose selection. After the elimination of both plasmids, the strains’ antibiotics resistances provided by the plasmids were absent, thus the plasmid-eliminated strains could not grow on the antibiotics-containing plates. All the strains were cultured at 37°C for 14 h on sucrose- or antibiotics-containing LB solid media. Using the primer pairs F3/R4 for *kgtP*-verification (F5/R6 for *xylF-*verification), the length of PCR production using *E. coli* BL21 genome DNA as template is about 2.3 kbp (2 kbp), containing about 1-kbp homologous arms and 1.3-kbp *kgtP* gene (1-kbp *xylF* gene), while the length of PCR productions using *E. coli* Δ*kgtP* (*E. coli* Δ*xylF*) genome DNA or homologous recombination related plasmid pACRISPR-sgRNA-HRkgtP (pACRISPR-sgRNA-HRxylF) as templates is about 1 kbp, implying gene *kgtP* (*xylF*) was deleted in corresponding strains.


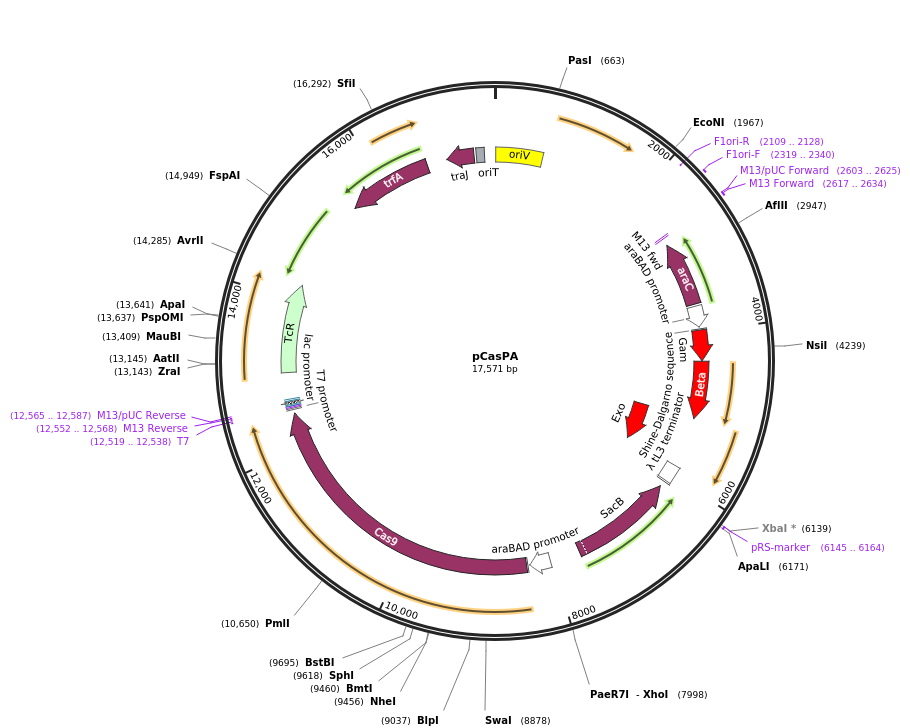


**Fig. S5 Map of plasmid pCasPA**


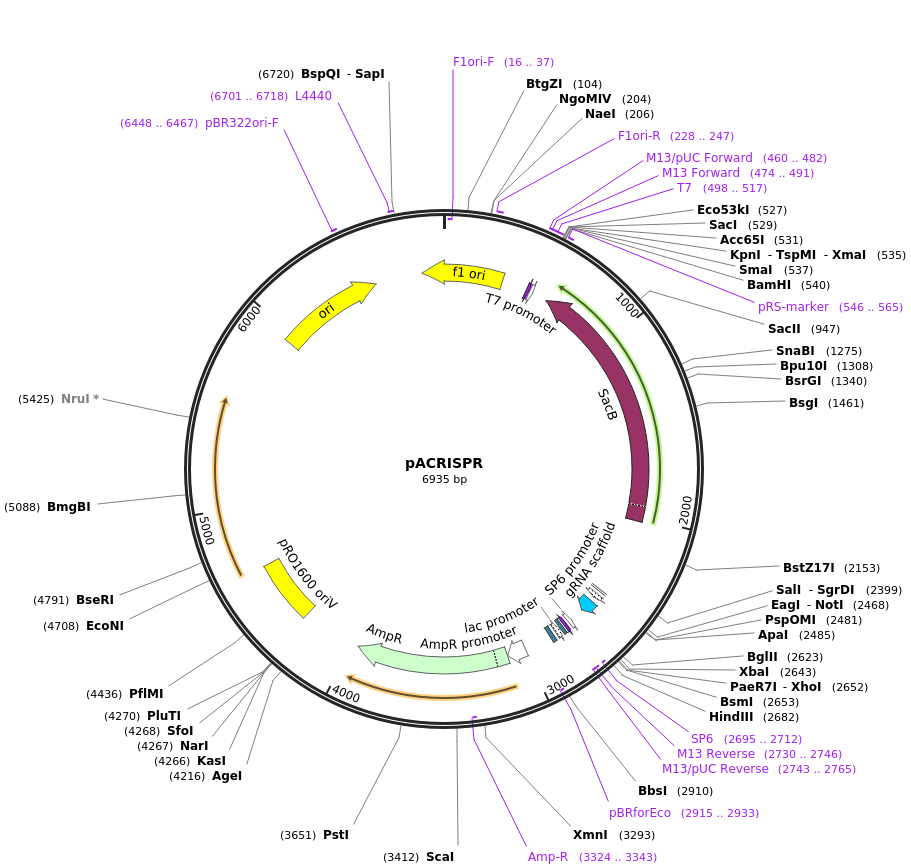


**Fig. S6 Map of plasmid pACRISPR**
